# Dissection of Pol II Trigger Loop Function and Pol II Activity–Dependent Control of Start Site Selection *In Vivo*

**DOI:** 10.1371/journal.pgen.1002627

**Published:** 2012-04-12

**Authors:** Craig D. Kaplan, Huiyan Jin, Ivan Liang Zhang, Andrey Belyanin

**Affiliations:** Department of Biochemistry and Biophysics, Texas A&M University, College Station, Texas, United States of America; University of Wisconsin, United States of America

## Abstract

Structural and biochemical studies have revealed the importance of a conserved, mobile domain of RNA Polymerase II (Pol II), the Trigger Loop (TL), in substrate selection and catalysis. The relative contributions of different residues within the TL to Pol II function and how Pol II activity defects correlate with gene expression alteration *in vivo* are unknown. Using *Saccharomyces cerevisiae* Pol II as a model, we uncover complex genetic relationships between mutated TL residues by combinatorial analysis of multiply substituted TL variants. We show that *in vitro* biochemical activity is highly predictive of *in vivo* transcription phenotypes, suggesting direct relationships between phenotypes and Pol II activity. Interestingly, while multiple TL residues function together to promote proper transcription, individual residues can be separated into distinct functional classes likely relevant to the TL mechanism. *In vivo*, Pol II activity defects disrupt regulation of the GTP-sensitive *IMD2* gene, explaining sensitivities to GTP-production inhibitors, but contrasting with commonly cited models for this sensitivity in the literature. Our data provide support for an existing model whereby Pol II transcriptional activity provides a proxy for direct sensing of NTP levels *in vivo* leading to *IMD2* activation. Finally, we connect Pol II activity to transcription start site selection *in vivo*, implicating the Pol II active site and transcription itself as a driver for start site scanning, contravening current models for this process.

## Author Summary

Transcription by multisubunit RNA polymerases (msRNAPs) is essential for all kingdoms of life. A conserved region within msRNAPs called the trigger loop (TL) is critical for selection of nucleotide substrates and activity. We present analysis of the RNA Polymerase II (Pol II) TL from the model eukaryote *Saccharomyces cerevisiae*. Our experiments reveal how TL residues differentially contribute to viability and transcriptional activity. We find that *in vivo* growth phenotypes correlate with severity of transcriptional defects and that changing Pol II activity to either faster or slower than wild type causes specific transcription defects. We identify transcription start site selection as sensitive to Pol II catalytic activity, proposing that RNA synthesis (an event downstream of many steps in the initiation process) contributes to where productive transcription occurs. Pol II transcription activity was excluded from previous models for selection of productive Pol II start sites. Finally, drug sensitivity data have been widely interpreted to indicate that Pol II mutants defective in elongation properties are sensitized to reduction in GTP levels (a Pol II substrate). Our data suggest an alternate explanation, that sensitivity to decreased GTP levels may be explained in light of Pol II mutant transcriptional start site defects.

## Introduction

Cellular DNA-dependent RNA polymerases likely balance fidelity in substrate selection with synthesis speed to achieve appropriate transcriptome content and regulation *in vivo*. In multisubunit RNA polymerases (msRNAP) from archaea, bacteria and eukaryotes, a highly conserved subdomain known as the trigger loop (TL) is critical for rapid catalysis and selection of correct substrates [1]–[9]. The TL is present in the largest subunit of eukaryotic Pol II, generally referred to as Rpb1 (Rpo21 in *Saccharomyces cerevisiae*), and the analogous β′ subunit of bacterial RNAP, and A″ subunit of archaeal RNAP.

Similarly to mobile domains of other classes of nucleic acid polymerases, the TL undergoes conformational changes in conjunction with the presence of an NTP substrate complementary to the DNA template (matched) in the msRNAP active site [1], [3]. These conformational changes are proposed to link TL-substrate interactions to preferential catalysis of correctly matched substrates over mismatched substrates. The TL can be observed in distinct conformations depending on the presence of matched NTP substrate, natural product polymerase inhibitors, and msRNAP-interacting proteins, underscoring its flexibility [1]–[3], [10]–[15]. In addition to effects on phosphodiester bond catalysis, the TL has been implicated in polymerase pausing, intrinsic cleavage of RNA and translocation [7]–[9], [16], [17].

**Figure 1:**
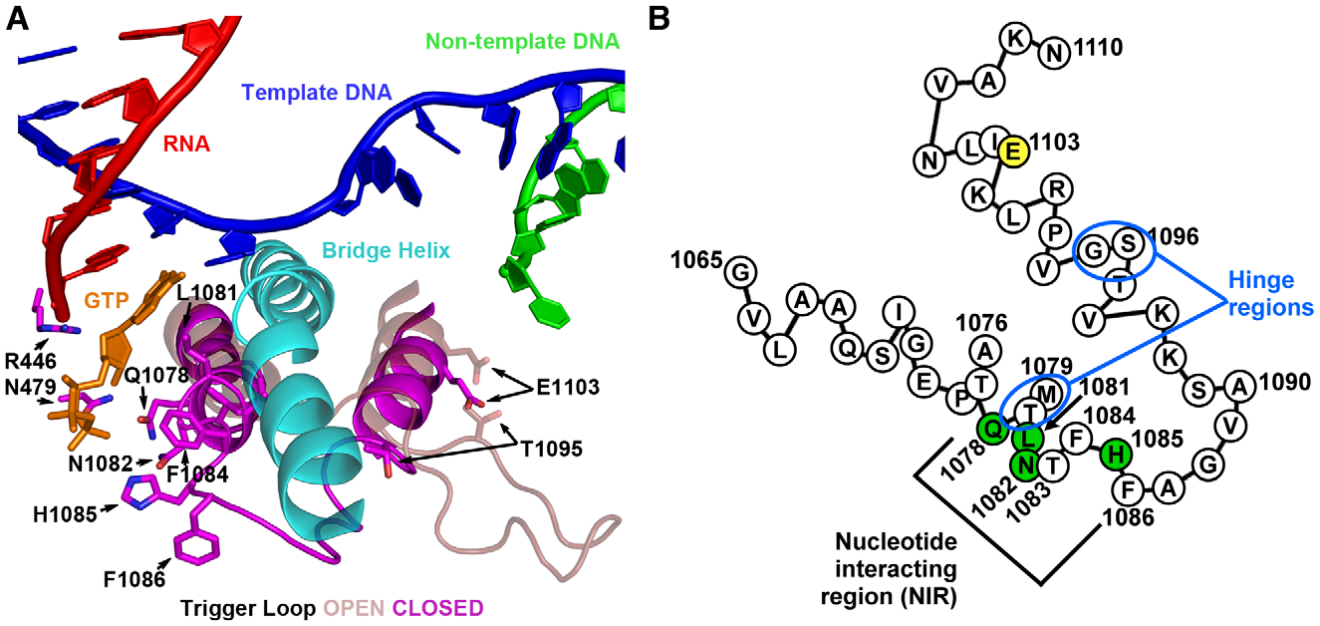
*S. cerevisiae* Rpb1 trigger loop conformations and sequence. A. Cartoon representation of “closed” Pol II TL in relation to nucleic acids, Rpb1 bridge helix and a matched GTP substrate from structure PDB 2E2H [1] overlaid with TL constrained in open conformation by TFIIS (not shown) from structure PDB 1Y1V [69]. Amino acids (all derived from Rpb1) adjacent to the matched GTP substrate are indicated by numbers and single-letter amino acid codes. This figure was created with Pymol [111]. B. Schematic of TL showing amino acid sequence in single-letter code, with positions of interest numbered, residues with direct contact to GTP substrate in structure 2E2H shown in green, and position 1103 shown in yellow. Two hinge regions, about which the TL appears to change conformation from the open to closed positions are indicated.

The TL comprises two mostly alpha-helical regions connected by a short loop (Figure 1A). Deletion or structural compromise of the TL in either *E. coli* (*Eco*) or *T. thermophilus* (*Tth*) strongly reduces catalytic activity, but to different extents depending on whether the substrate is matched to the template or not [3]–[5], [7]. Two specific regions of the TL appear important for control of TL function. First, conserved residues in the nucleotide interacting region (NIR) recognize specific features of matched NTP substrates and work in conjunction with non-TL residues, Rpb1 N479 and R446, positioned for interaction with hydroxyl moieties on the ribose of matched NTPs [1], [3](Figure 1A). Residues in the NIR showing NTP interactions in *S. cerevisiae* and *Tth* substrate-bound structures are (using *S. cerevisiae* Rpb1/Rpo21*^Eco^* ^β*′/Tth*β*′*^ numbering) Gln1078^Gln930/Gln1236^, Leu1081^Met932/Met1238^, Asn1082^Arg933/Arg1239^ and His1085^His936/His1242^.Second, in all kingdoms of life, substitutions in or near the helix distal to the NIR alter elongation rate, in some cases increasing elongation rate relative to WT (“superactivity”), [2], [16], [18], [19]. These substitutions may alter dynamics of TL movement between the substrate-interacting conformation and other conformations because they are adjacent to the hinge region in the C-terminal TL helix (another hinge is apparent in the N-terminal TL helix)(Figure 1B) [2], [9].

NIR residues of the *Eco* and *Tth* RNAPs have different degrees of contribution to catalytic activity, with individual *Eco* residue substitutions having smaller effects on activity than homologous substitutions in *Tth*, underscoring the diversity of TL residue functions in msRNAPs [5], [7]. A conserved histidine within the TL NIR has been proposed to have an important or essential catalytic role [1]. Many classes of structurally distinct nucleic acid polymerases utilize a basic residue located at a position in the active site analogous to that of the TL histidine and these residues function as a general acid in the catalytic cycle of the polymerase [20]. While clearly important for RNAP catalysis, the TL histidine appeared not to be functioning as a general acid based on pH-dependence curves for bacterial RNAPs [5], [7]. Experiments in the different bacterial systems show that a TL methionine residue (equivalent to *S. cerevisiae* Leu1081) packs against a base-paired NTP in the active site and has a greater contribution to activity than Arg*^Eco^* ^933/*Tth*1239^ or His*^Eco^* ^936/*Tth*1242^, which interact with the triphosphate moiety of matched substrates [5], [7].

Genetic identification of mutants with substitutions in the TL region, first in *S. cerevisiae* Pol II, then in *Eco* RNAP, demonstrated that alteration of the TL could alter transcription *in vivo* [16], [18], [21]–[23]. How changes in TL function or msRNAP catalysis alter transcription *in vivo* are not well understood and to what extent polymerase activity defects may be tolerated *in vivo* is not clear. In *S. cerevisiae*, drugs that target nucleotide synthesis pathways such as mycophenolic acid (MPA, targets GTP synthesis) [24] and 6-azauracil (6-AU, targets UTP and GTP synthesis) [25], [26] have been shown to cause alterations in gene expression *in vivo* [27]–[30]. A large number of Pol II transcription-related mutant strains show altered sensitivities to these drugs, leading to the broadly utilized interpretation that these drugs are elongation inhibitors and that sensitivity to them suggests defective Pol II elongation [18], [31]–[54]. Notwithstanding the large number of mutants sensitive to these drugs that have no known transcriptional role, many MPA-sensitive mutants alter transcription of the gene *IMD2* [35], [38], [47], which encodes an MPA-resistant form of IMPDH, the enzymatic activity targeted by the drug [55], [56]. This gene-specific transcription defect is not always considered when interpreting mutant phenotypes. Intriguingly, *IMD2* transcription involves a switch between upstream transcription start sites and downstream productive start sites that differ in initiating NTPs (upstream: GTP, downstream: ATP) leading to the proposal that the initiation process for these different classes of transcript stems from GTP levels being sensed directly by Pol II [57], [58].

The eukaryotic Pol II system provides an excellent model for *in vivo* studies of how the TL functions in transcription. Because nuclear transcription in eukaryotes is segregated among three essential polymerases instead of one, as in bacteria and archaea, strong defects may be more tolerated in Pol II than bacterial or archaeal RNAPs *in vivo*. We utilized extensive site-directed mutagenesis of the Pol II TL coupled with genetic screening to identify viable substitutions in TL NIR residues and substitutions conferring conditional growth phenotypes. We then used biochemical characterization together with a number of *in vivo* genetic and molecular phenotypes to probe the contributions of critical TL residues to transcription *in vivo*. We determined the relationship between NIR residues and superactivating TL substitutions and found that superactivating substitutions were mostly mutually suppressive with loss-of-function substitutions within the NIR in genetic and biochemical assays. These results indicated that NIR residues were not bypassed by superactivating substitutions and were still required for Pol II activity. Using a series of TL mutants that define a continuum of *in vitro* elongation rates, we demonstrated that a number of *in vivo* phenotypes correlated closely with Pol II activity *in vitro*. We provide support for models proposing that *IMD2* transcription is directly sensitive to Pol II activity, therefore explaining the MPA-sensitivity of superactive Pol II mutants, which otherwise might have been expected to be resistant to reduced GTP levels due to increased elongation activity. Finally, we determined that start site selection at a number of other genes was similarly sensitive to alteration in Pol II activity leading to a new model for transcription-dependent polarity of start site selection in *S. cerevisiae*.

## Results

### Genetic Analyses of Rpb1 TL Mutants *In Vivo*

We have undertaken an extensive genetic dissection of the Pol II TL: specifically, we examined the contribution of TL residues to Pol II function, and how Pol II catalytic activity relates to transcription *in vitro* and *in vivo*. To examine a large number of Pol II mutants *in vivo*, we employed a yeast strain containing a deletion of the endogenous copy of *RPO21* (the gene encoding Rpb1, which we henceforth refer to as *RPB1*) with *RPB1* activity complemented by a low copy *CEN* plasmid containing *RPB1* genomic DNA or mutant variants, allowing expression from the native *RPB1* promoter. Site-directed mutagenesis was focused on TL NIR residues to identify viable substitutions and was combined with existing *rpb1* TL alleles identified in genetic screens (Text S1, genetic screens to be described elsewhere). For any viable mutants, alteration in transcription *in vivo* was measured with two phenotypic reporters for transcriptional defects, the *lys2-128∂* [59] and *gal10*Δ*56* alleles [60], [61](Figure S1). These reporters are modulated by a number of transcription elongation factors and Pol II mutants with known transcription defects [2], [60], [62]–[66].

Previous analyses indicated that some TL substitutions cause increases in Pol II elongation rate *in vitro* (“superactivity” or gain of function (GOF))(*e.g.* E1103G) [2], [9], [18]; some of these increases in rates for misincorporation were greater than in rates for incorporation of templated NTPs, indicating possible loss of fidelity (F1084I, E1103G) [2], [9]. Other substitutions confer reduced elongation rate (loss of function (LOF))(H1085Y, F1086S) [2]. *In vitro* activity appeared to correlate with *in vivo* phenotypes for the few mutants examined [2]. Suppression of a growth defect on media lacking lysine conferred by *lys2-128∂* (the Spt^−^ phenotype) was observed for known TL GOF alleles and neighboring substitutions [2]. Lysine auxotrophy of *lys2-128∂* relates to defective *LYS2* transcription caused by a retrotransposon insertion in *LYS2* [59]. Some of these GOF alleles are also sensitive to MPA or 6-AU, while the Spt^−^ phenotype and strong MPA sensitivity have not been observed among known TL LOF alleles [2], [18]. Both known LOF and GOF alleles confer alteration of growth phenotypes relating to transcriptional interference at the *GAL10-GAL7* locus when polyadenylation and termination at *GAL10* are compromised in the *gal10*Δ*56* allele [2].

**Figure 2:**
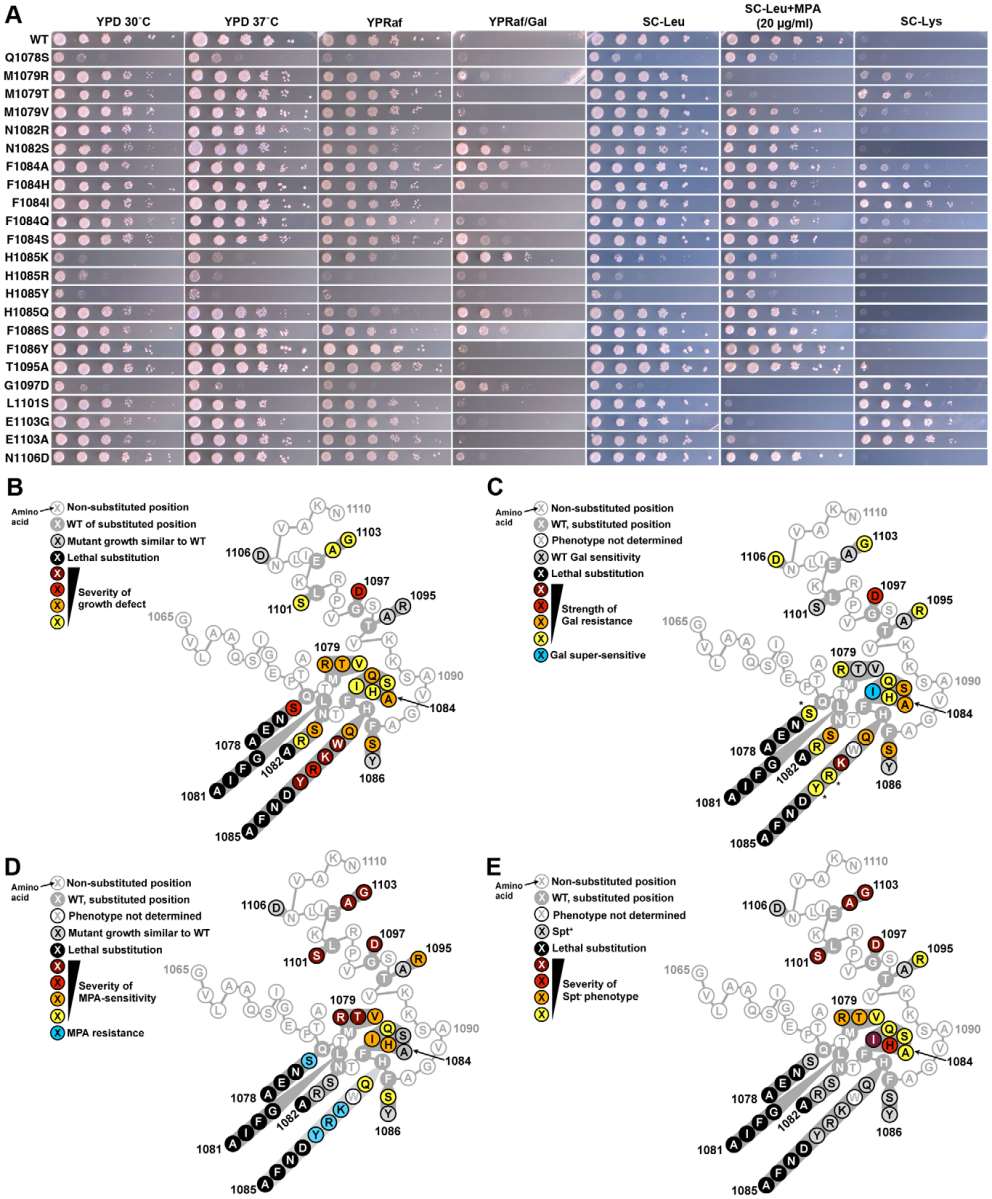
Genetic analyses of Pol II TL single substitution mutants. A. 10-fold serial dilutions of saturated cultures of Pol II TL mutant strains plated on different media. YPD is rich medium with dextrose as a carbon source. YPRaf is rich medium with raffinose as a carbon source. YPRaf/Gal has both raffinose and galactose, allowing assay of *gal10*Δ*56*-dependent galactose toxicity phenotypes. SC-Leu is defined, complete medium lacking leucine. MPA was added to this medium (SC-Leu+MPA) to 20 µg/ml final concentration, showing that a number of Pol II mutants are sensitive to this drug. SC-Lys is defined, complete medium lacking lysine, and detects the Spt^−^ phenotype (Lys^+^) for strains containing *lys2-128∂*. WT strains grow robustly on most media, but will not grow on SC-Lys when *lys2-128∂* is present and grow very poorly on YPRaf/Gal when *gal10*Δ*56* is present. Mutant-dependent transcriptional phenotypes allow modulation of these specific growth defects. B. Schematic of TL (as diagrammed in Figure 1B) showing distribution of viable substitutions with their growth defects on YPD as well as lethal substitutions. All mutants were examined in the background of an Rpb1 T69 change, which is the allele present in the S288C reference genome as well as being normally found in our strains, representing a distinction for the subset of mutants described previously [2](see Materials and Methods, Text S1 and note concerning viability of Q1078S). Scoring of phenotypic strength is based on visual inspection of (A). C. Distribution of *gal10*Δ*56* suppression (Gal resistance) or enhancement (Gal super-sensitivity) among TL substitutions. Scoring of phenotypic strength is based on visual inspection of (A). *Indicates strength of *gal10*Δ*56* suppression is likely underestimated due to confounding generic growth defects. D. Distribution of MPA phenotypes among TL substitutions. Scoring of phenotypic strength is based on visual inspection of (A). **E.** Distribution of *lys2-128∂* suppression (Spt^−^ phenotype scored as Lys^+^) among TL substitutions. Scoring of phenotypic strength is based on visual inspection of (A).

In our current study, we found that the Spt^−^ phenotype was concentrated in residue substitutions proximal to the two TL “hinges” (Figure 2A, 2E, see Figure 1B for positions of hinges). Growth defects were conferred by substitutions throughout the TL (Figure 2A, 2B). We found that MPA-sensitivity (MPA^s^) and Spt^−^ phenotypes generally co-occurred although the relative strength of the two phenotypes varied among mutants (Figure 2A, 2D, 2E). Finally, we found that the strongest suppressors of *gal10Δ56*, with the exception of G1097D, were in TL NIR residues and were mostly Spt^+^ or MPA-resistant (MPA^r^), indicating a distinction between these phenotypes (Figure 2C). Taken together, these conditional plate phenotypes behave as sensitive readouts for likely complex sets of overlapping or distinct transcriptional defects *in vivo*.

### *In Vitro* Transcription Elongation Rates Correlate with *In Vivo* Growth Defects and Plate Phenotypes

**Figure 3:**
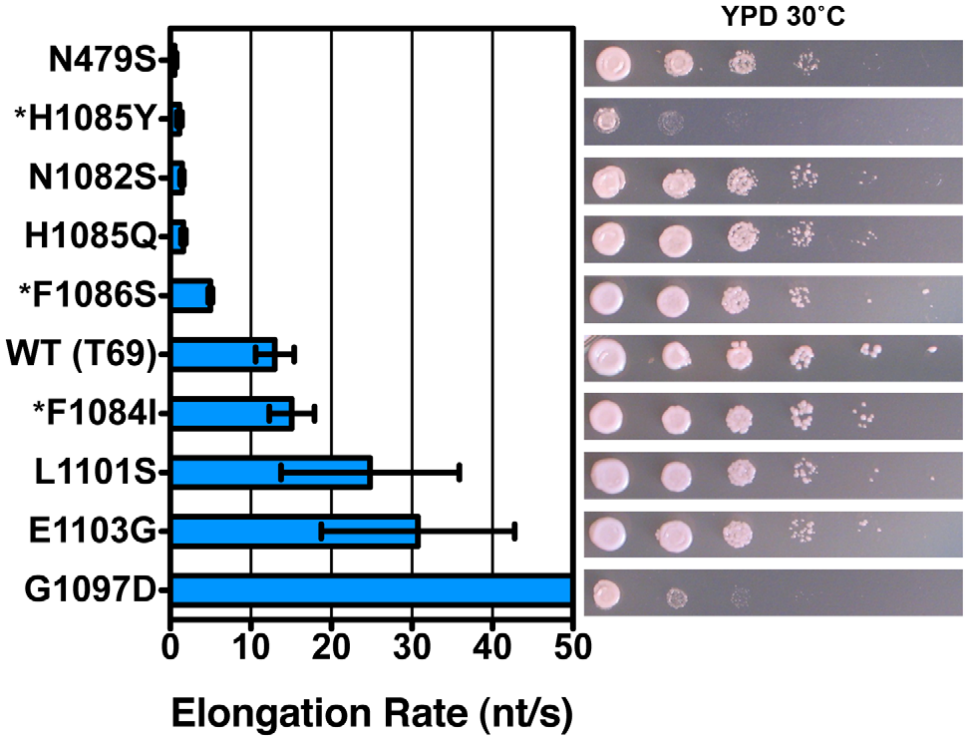
Pol II TL substitution mutants show a wide range of elongation rates *in vitro*. Pol II TL single substitution elongation rates as determined *in vitro* using reconstituted Pol II elongation complexes with synthetic oligonucleotides and an RNA primer (Materials and Methods). Raw data for some mutants (*) are from [2] and are shown for comparison. Plotted data are maximal elongation rates as determined by non-linear regression of elongation rates determined for different NTP substrate concentrations, and error bars indicate the range of the 95% confidence intervals (Figures S4, S5). Growth on rich medium of mutants is shown in the right panels for comparison with elongation defects (from Figure 2).

As TL substitutions causing the Spt^−^ phenotype included those with known TL GOF phenotypes (F1084I, E1103G) [2], we asked if our plate phenotypes were predictive of biochemical phenotypes. Analysis of TL substitutions in an *in vitro* transcription assay using reconstituted Pol II elongation complexes indicated a good correlation between elongation rate defect *in vitro* and growth defect *in vivo* (Figure 3, Figures S4 and S5). We note that we did not detect strong expression differences for tested mutant Rpb1 proteins (Figure S6). We previously showed that LOF substitution H1085Y and a putative GOF substitution (G1097D) caused severe growth defects *in vivo* [2]. In this study we find that these two mutants also represent extremes of Pol II activity defects, but interestingly at different ends of the activity spectrum. Biochemical analysis of Spt^−^ and MPA^s^ G1097D and L1101S shows that they are GOF substitutions conferring increased maximal elongation rate *in vitro*. In fact, G1097D elongates too quickly for accurate measurement in our short template run off assay (Figure 3)(Figures S4 and S5). We evaluated a number of other TL substitutions and found that WT elongation rate *in vitro* correlates with robust growth *in vivo*, and that the greater the deviation from WT for elongation rate *in vitro*, the greater the deviation from WT growth *in vivo* (Figure 3, right panel). We note that the non-TL LOF substitution, N479S, deviates slightly from this relationship as it has the most severe *in vitro* defect but has a less severe growth defect. This could be consistent with a factor(s) functioning *in vivo* that can compensate for the biochemical defect caused by the N479S substitution but not the H1085Y substitution; for example, a hypothetical factor that may work through the TL.

### Role of E1103-T1095 Interactions in Superactivation of E1103G

For msRNAPs from each kingdom of life, GOF substitutions have been reported in TL residues distal to the NIR and proximal to the C-terminal “hinge” region [2], [9], [16], [18], [19], which is the portion of the TL that changes conformation from open to the closed NTP-interacting orientation (Figure 1). There is evidence that the Pol II GOF substitution E1103G promotes a closed TL state [9]. An increased frequency or duration of this state could have consequences for catalysis (increased activity with all substrates and net increase in misincorporation) and translocation (reduced translocation rate likely due to stabilization of closed state after catalysis). The E1103 sidechain interacts with the T1095 sidechain and makes backbone contacts with K1112 in elongation complex crystal structures where the TL is in the open conformation or constrained by other factors [67]–[69]. Loss of this E1103-T1095 interaction has been posited to explain the effect of E1103G on Pol II transcription [9]. We found that the T1095A substitution was phenotypically indistinguishable from wild type (WT), indicating that loss of T1095-E1103 contacts are not responsible for E1103G growth alteration or, most likely, biochemical superactivity (Figure 2). T1095R, presenting a much longer sidechain that might disrupt “out conformation” hinge folding, only confers weak MPA^s^, Spt^−^ and Gal^r^ phenotypes, consistent with C-terminal hinge function or TL dynamics being controlled more strongly by distal residues or other contacts of E1103 (Figure S3).

### Combinatorial Analyses of TL Substitutions Reveal Functional Distinctions between Residues with Similar Single-Mutant Behavior

**Figure 4:**
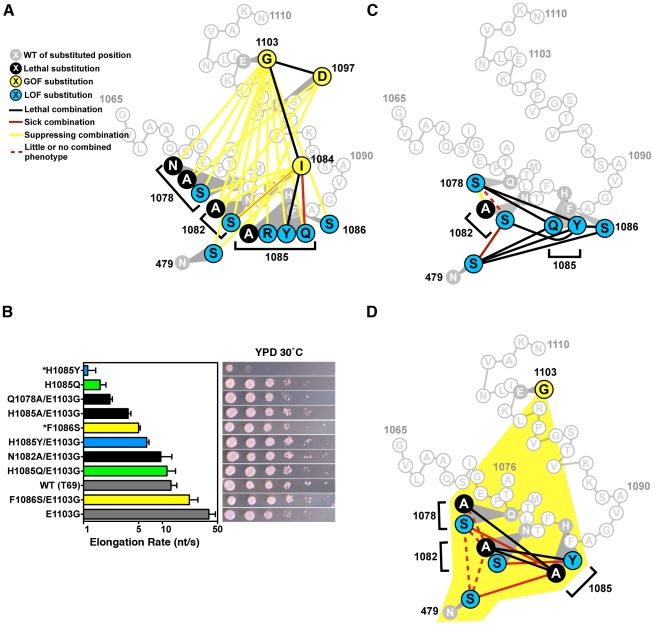
Combination of TL substitutions alters *in vivo* growth and *in vitro* biochemical phenotypes. A. GOF-GOF and GOF-LOF genetic interactions among *rpb1* TL and other substitutions. GOF (yellow) and LOF (blue) classifications were determined by biochemical and genetic phenotypes of single substitution mutants, with lethal single substitutions shown in black. Double mutant phenotypes are illustrated as the colored lines connecting colored nodes of particular single mutants. Schematic of TL sequence and orientation is as in Figure 1B. Data were compiled from Figure S2, Figure S3 and Table 1. N1082S/F1084I shows a complex interaction with exacerbation of growth defects on rich medium but mutual suppression of other conditional plate phenotypes. B. *In vitro* elongation rates determined as in Figure 3 for select Pol II TL double mutant enzymes. Single mutants and their relevant E1103G double mutants are indicated by color-coding: F1086 and F1086/E1103G, yellow; H1085Q and H1085Q/E1103G, green; H1085Y and H1085Y/E1103G, blue. Black bars indicate double mutants with E1103G for which the counterpart single substitution mutant is inviable (Q1078A, N1082A, H1085A). Single mutant data are from Figure 3. Raw data for some mutants (*) are from [2] and are shown for comparison. Error bars indicate the ranges of the 95% confidence intervals. Note that *x*-axis is logarithmic scale. Growth on rich medium of mutants is shown in the right panels for comparison with elongation defects (from Figure 2, Figure S2). C. Interaction diagram showing phenotypes of pairwise combinations of *rpb1* LOF alleles (legend shown in (A)). D. Interaction diagram illustrating genetic interactions between pairs of Rpb1 substitution mutants each in the presence of the E1103G substitution (yellow shading encompassing all relevant residues, legend shown in (A)).

We next wished to probe genetic relationships between TL GOF and LOF substitutions, particularly those between TL hinge residues and TL NIR residues, as these relationships might provide mechanistic insight into TL function (Figure 4A, Figure S2, Figure S3, Text S1). Surprisingly, by combining TL GOF with TL NIR substitutions, we found that either E1103G or G1097D GOF mutations were able to suppress lethality of many, but not all, inviable (LOF) single substitution NIR mutants (Figure 4A, Figure S2, Table 1). Examination of double mutant plate phenotypes provided more evidence of mutual suppression in E1103G-NIR mutant combinations because Spt^−^ and MPA^s^ phenotypes of E1103G were suppressed together with moderate Gal^r^ phenotypes of individual NIR substitutions (Figure S2). In contrast, tested pairwise combinations of genetically and biochemically similar GOF substitutions, F1084I, G1097D and E1103G resulted in lethality (Figure 4A). Additionally, combination of viable LOF NIR-residue substitutions with the GOF substitution E1103G resulted in mutual suppression of conditional plate phenotypes and growth defects (Figure S2).

**Table 1:** Mutational analysis of the Pol II TL.

Substitution	Phenotype	Note
Q1078A	Inviable	
Q1078N	Inviable	[2]
Q1078E	Inviable	
L1081I	Inviable	
L1081A	Inviable	
L1081G	Inviable	
L1081F	Inviable	
N1082A	Inviable	
H1085A	Inviable	[2]
H1085D	Inviable	
H1085N	Inviable	
H1085F	Inviable	[2]
N479S/N1082S	Viable	Figure S3
N479S/F1084I	Viable	Figure S3
N479S/H1085Q	Inviable	Double mutant lethality
N479S/H1085Y	Inviable	Double mutant lethality
N479S/F1086S	Inviable	Double mutant lethality
Q1078S/N1082A	Viable	Q1078S suppresses N1082A lethality, Figure S3
Q1078S/N1082S	Viable	Figure S3
Q1078S/F1084I	Viable	Figure S3
Q1078S/H1085Y	Inviable	Double mutant lethality
Q1078S/H1085Q	Inviable	Double mutant lethality
Q1078S/H1085A	Inviable	
Q1078A/N1082A	Inviable	
N1082S/F1084I	Viable	Figure S3
N1082S/H1085Y	Inviable	Double mutant lethality
N1082A/F1084I	Viable	Figure S3
N1082A/H1085Y	Inviable	Double mutant lethality
F1084I/H1085Q	Viable	Figure S3
F1084I/H1085Y	Inviable	Double mutant lethality
F1084I/E1103G	Inviable	Double mutant lethality
G1097D/E1103G	Inviable	Double mutant lethality
N479S/Q1078S/E1103G	Viable	Figure S3
N479S/N1082A/E1103G	Inviable	Triple mutant lethality
N479S/H1085A/E1103G	Viable	Figure S3
N479S/F1084I/E1103G	Viable	Suppression of F1084I/E1103G lethality, Figure S3
Q1078S/N1082A/E1103G	Viable	Figure S3
Q1078A/N1082A/E1103G	Viable	Figure S3
Q1078A/H1085Y/E1103G	Inviable	Triple mutant lethality
Q1078A/H1085A/E1103G	Inviable	Triple mutant lethality
Q1078S/H1085A/E1103G	Viable	Figure S3
N1082S/F1084I/E1103G	Viable	Figure S3
N1082A/F1084I/E1103G	Viable	Figure S3
N1082S/H1085Y/E1103G	Viable	Figure S3
N1082A/H1085Y/E1103G	Inviable	Triple mutant lethality
N1082A/H1085A/E1103G	Inviable	Triple mutant lethality
F1084I/H1085Y/E1103G	Inviable	H1085Y cannot suppress F1084I/E1103G
Q1078S/N1082A/H1085Y/E1103G	Viable	Figure S3
A1076V/Q1078S/N1082A/H1085Y/E1103G	Viable	Figure S3
A1076V/Q1078A/N1082A/H1085Y/E1103G	Viable	Figure S3
N1082A/H1085A/G1097D	Inviable	
ΔTL Tip (Δ1077-GG-1087)	Inviable	Deletion of TL residues 1077–1087, insertion of Gly-Gly linker
ΔTL Tip/E1103G	Inviable	E1103G cannot suppress deletion of TL NIR

Abilities of single and multiply substituted Pol II variants to complement the inviability of a deletion of *RPO21/RPB1* are illustrated, along with notes on mutant behavior and relevant figures.

If observed plate phenotypes directly relate to elongation rate, as suggested by our analysis of single substitution mutations (Figure 3), the observations above predict that the double mutants' *in vitro* elongation rates might be closer to WT than those of the single substitution mutants. We therefore measured elongation rates of doubly substituted TL mutants to further examine the relationships between GOF and LOF variants (Figure 4B)(Figures S4 and S5). We found that combination of E1103G and individual viable LOF substitutions F1086S, H1085Q or H1085Y resulted in enzymes with activity intermediate between the relevant singly substituted enzymes. Combination of E1103G individually with the lethal single substitutions Q1078A, N1082A or H1085A resulted in viable strains and Pol II enzymes with activity reduced compared to E1103G alone. The fold enhancement of viable LOF substitutions F1086S, H1085Q and H1085Y by E1103G was roughly similar in each case.

To determine if TL NIR residues function individually or in a coupled fashion, we constructed pairwise combinations using TL NIR LOF alleles and a substitution in the TL-adjacent Rpb1 NTP-interacting residue, N479S (Figure 4C). We observed lethality when H1085 substitutions were combined with substitutions in other TL NIR residues or with N479S, consistent with H1085 functioning non-redundantly or distinctly from other TL NIR residues or N479 in the Pol II nucleotide addition cycle. Surprisingly, when we examined combinations among N479, Q1078, and N1082 substitutions we did not observe lethality (Figure 4C). In fact, we observed suppression or epistasis between Q1078 and N1082 substitutions. These observations suggested a functional distinction between N-terminal TL NIR residues (Q1078, N1082) and H1085. Viable substitutions in these residues maintain sidechains that might still confer some interaction between the TL and NTPs, however alanine substitutions in key residues Q1078, N1082, and H1085 were inviable and could not be directly assessed genetically. Because all LOF TL residues, including these lethal substitutions, were suppressed by E1103G, we examined triple mutants that contained pairwise substitutions in the TL NIR in the background of E1103G (Figure 4D). These triple mutant combinations revealed similar functional distinctions between two sets of Pol II active site residues, with the set of residues containing N479, Q1078 and N1082 being distinct from H1085. These results may be interpreted in light of structural information and have implications for the mechanism of TL function (see Discussion).

### *In Vivo* Transcription Defects of Pol II TL Mutants

**Figure 5:**
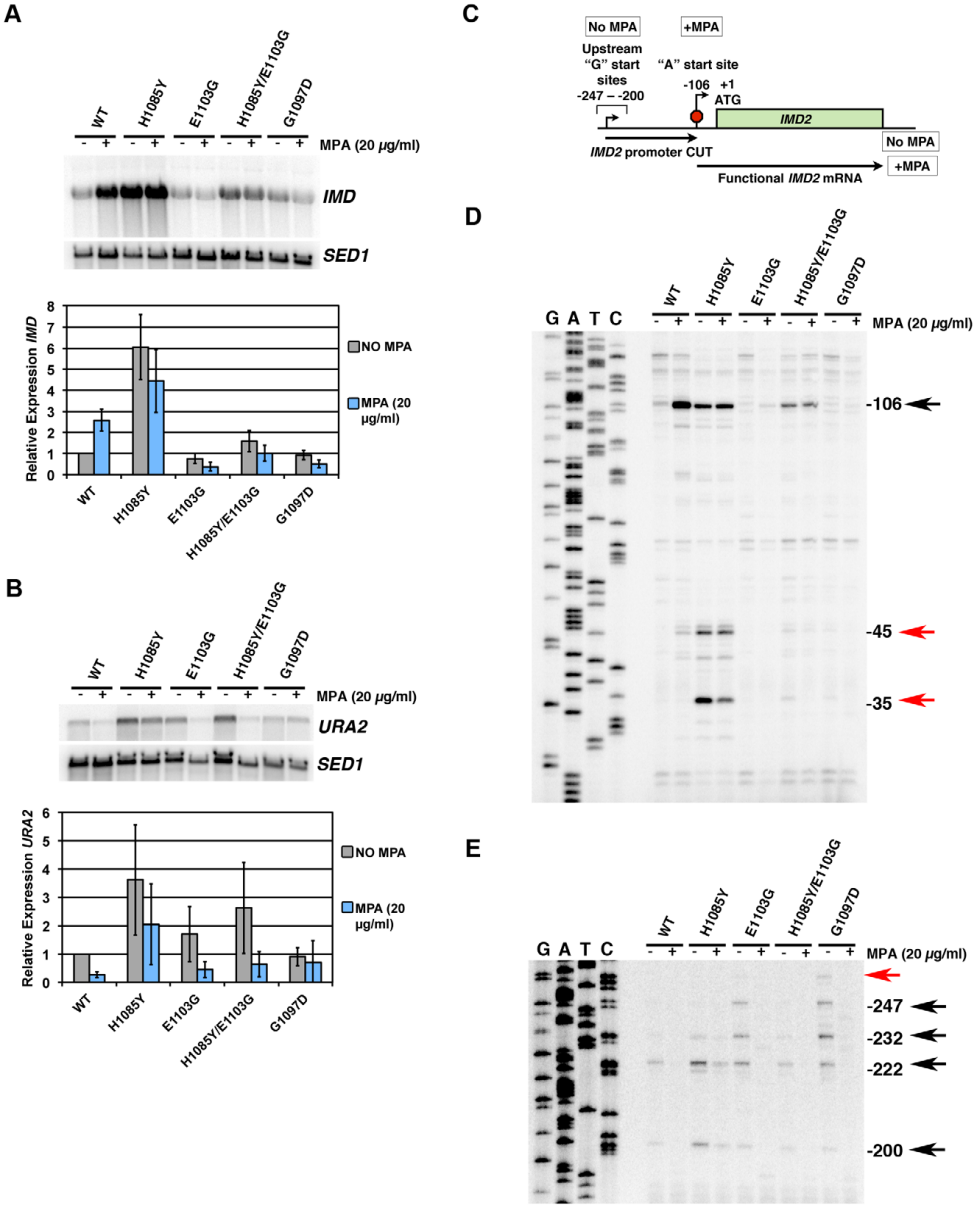
Pol II TL mutants alter transcription of *IMD2* and *URA2*, genes required for GTP and UTP synthesis, respectively. A. Northern blotting for different Pol II mutants in the presence or absence of 20 µg/ml MPA (2 hours treatment) for expression of *IMD* gene(s) using *IMD2* DNA probe (top). *SED1* was probed as a loading control. Values for *IMD* normalized to the WT ratio of *IMD/SED1* are shown in the graph and represent the mean relative expression for *IMD* transcript(s) +/− the standard deviation for at least three independent experiments. B. Northern blotting for *URA2* expression in the presence or absence of 20 µg/ml MPA (2 hours treatment) (top). *SED1* was probed as a loading control. Values for *URA2* normalized to the WT ratio of *URA2/SED1* are shown in the graph and represent the mean relative expression for *URA2* +/− the standard deviation for at least three independent experiments. C. Schematic of *IMD2* gene transcription in the absence or presence of MPA. *IMD2* is not functionally expressed in the absence of MPA or low GTP because upstream transcriptional starts are terminated at a terminator element (stop sign) that can be bypassed by utilization of downstream start sites. D. Primer extension analysis for downstream *IMD2* start site usage in Pol II TL mutants in presence or absence of 20 µg/ml MPA (2 hours treatment). Numbers on right indicate position of RNA terminus relative to the A of the *IMD2* ATG codon. Sequence ladder on right is derived from the primer used in (E) and has been cross-referenced with the primer used here. E. Primer extension analysis for upstream *IMD2* start site usage in Pol II TL mutants as in (D). Numbers on right indicate position of RNA terminus relative to the A of the *IMD2* ATG codon. Sequence ladder on right is derived from the same primer used. The examples in D and E are representative of at least three independent experiments.

To examine our Pol II TL mutants for *in vivo* transcription defects, we analyzed expression of *IMD* genes and *URA2* (Figure 5A, 5B), which function in GTP and UTP synthesis, respectively. Expression of one *IMD* gene, *IMD2*, and *URA2*, have been linked to GTP or UTP levels, respectively, possibly directly through alteration of Pol II function by changes in levels of these transcription substrates [57], [58], [70], [71]. Expression of *IMD2* is required for WT yeast to be resistant to the drug MPA [46], [56]. *IMD2* transcripts are increased in the presence of MPA due to reduced GTP levels that result from inhibition of MPA-sensitive Imd2 homologs, Imd3 and Imd4 [55], [56]. A number of transcription mutants are defective for *IMD2* upregulation upon MPA treatment [35], [38], [47] while some Pol II mutants have been reported to show constitutive expression of *IMD2* [18], [57]. As *IMD2* has a high level of sequence similarity to the pseudogene *IMD1* and to *IMD3* and *IMD4*, and because of possible cross-hybridization [56], we do not specify that observed changes in our Northern blotting are solely due to *IMD2* changes. However, it has been demonstrated that the majority of MPA-induced *IMD* expression comes from *IMD2* [56].

We observed a constitutive upregulation of *IMD* transcripts in LOF mutant H1085Y and loss of MPA-responsiveness in GOF mutants E1103G and G1097D as well as in the H1085Y/E1103G double mutant (Figure 5A). E1103G appeared to have reduced expression of *IMD* genes in the absence of MPA treatment, and importantly, E1103G and G1097D showed reduced *IMD* expression upon MPA treatment. These results are consistent with the MPA-sensitivity of E1103G and G1097D and the apparent MPA-resistance of H1085Y (Figure 2) [2]. They also indicate that defects in *IMD* expression may fully explain the MPA-sensitivity of Pol II TL mutants and need not relate to global elongation defects due to reduced GTP levels, which is the commonly invoked mechanism for MPA-sensitivity. The results, however, are consistent with a model in which *IMD2* regulation occurs directly through Pol II-nucleotide sensing.

Activation of *URA2* has also been proposed to result from a Pol II-dependent NTP-sensing event because a class of Pol II mutants causes constitutive *URA2* expression [70], [71]. *URA2* is normally controlled by a change in start site selection from upstream start sites, which produce transcripts that are prematurely terminated and degraded, to downstream start sites that allow production of full-length mRNA. The Pol II mutants that lead to *URA2* expression are located in the “switch 1” region of Rpb1 [72], near a residue linked to start site selection [73]. We reasoned that if *URA2* were directly responsive to Pol II-sensing of UTP levels, then a Pol II TL LOF mutant should also show constitutive upregulation of *URA2*, as a reduction of Pol II activity through TL defects should mimic reduction in substrate levels. We found that H1085Y caused *URA2* to be upregulated (Figure 5B). GOF TL mutants E1103G and G1097D were closer to WT for *URA2* expression, while H1085Y/E1103G showed an increase in *URA2* expression. Furthermore, *URA2* was not responsive to MPA treatment, an expected result if *URA2* were specifically responsive to UTP depletion as opposed to GTP depletion.

*IMD2*, like *URA2*, is regulated by a shift in start sites from upstream starts that lead to premature termination, to downstream starts upon nucleotide limitation [57], [58]. The start site changes in *IMD2* are proposed to relate to concentration of initiating NTP, with upstream starts initiating with GTP, whereas under GTP-limiting conditions, the major downstream start initiates with ATP (Figure 5C). We examined this phenomenon in Pol II TL mutants by primer extension (Figure 5D, 5E). Examination of downstream start sites at *IMD2* showed that the major ATP start site at −106 from the *IMD2* start codon is induced by MPA treatment in WT cells (Figure 5D) [74]. TL LOF H1085Y showed constitutive expression from this start regardless of MPA treatment, and, interestingly, apparent usage of downstream cryptic starts at approximately −35 and −45. Although mRNA processing in theory could generate these shorter new transcripts, it is unlikely as processed transcripts lacking a 5′-cap should be fairly unstable. In contrast, for TL GOF mutants E1103G and G1097D little or no usage of the −106 start was observed, regardless of MPA treatment, consistent with MPA-sensitivity of these strains and Northern blotting showing no MPA induction of *IMD* transcripts. In the H1085Y/E1103G mutant that showed mutual suppression of respective single mutant phenotypes but reduced activity from WT *in vitro*, we observed constitutive usage of the −106 start, but at apparent lower levels than H1085Y. In addition, we observed MPA-dependent loss of upstream start site usage in this set of mutants, suggesting maintained responsiveness to MPA-effects for these starts, even though H1085Y showed constitutive use of downstream starts. In contrast to the observed H1085Y usage of new −35 and −45 downstream start sites (Figure 5D), we observed increased E1103G and G1097D usage of upstream starts relative to WT (Figure 5E), suggesting altered start site selection in Pol II TL mutants.

### Activity-Dependent Control of Pol II Start Site Selection *In Vivo*

**Figure 6:**
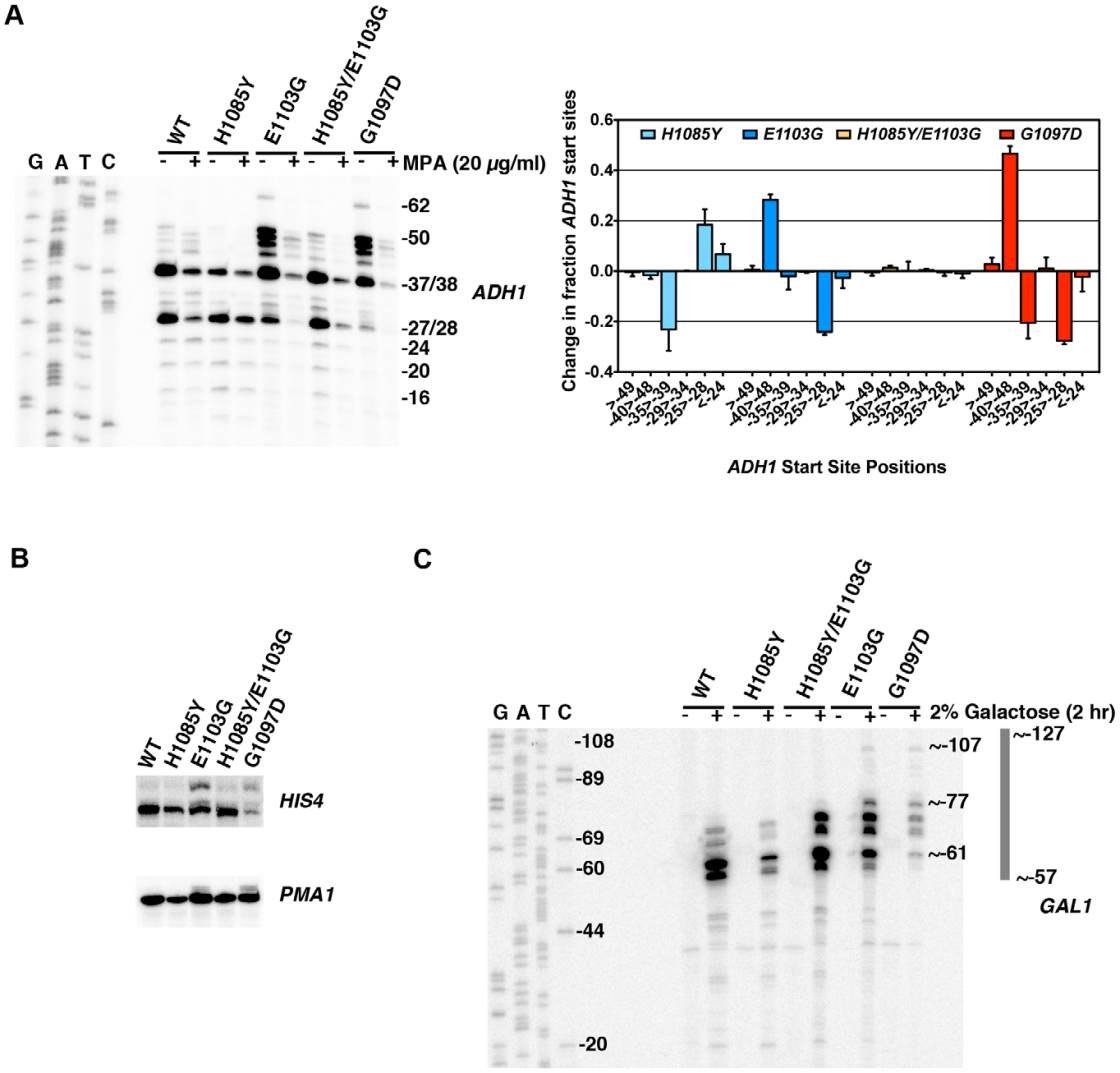
Pol II TL contributes to start site selection at a number of genes. A. Primer extension analysis of RNA 5′-ends at *ADH1* in Pol II TL mutants. Sequence ladder at left is derived from primer used in Figure 5E but has been cross-referenced with *ADH1* primer used for primer extension here. Numbers indicate positions of putative start sites in relation to the *ADH1* ATG (A is +1). Right panel quantifies start site usage at *ADH1* as determined by primer extension with a radiolabeled oligo. Signals in each lane were divided into bins based on positions of start sites relative to *ADH1* ATG sequence (A is +1) and normalized to total signal per lane. WT start site fractions were subtracted from mutant start site fractions for particular regions to determine the relative alteration in start site distribution in Pol II mutant strains. A negative value indicates that the mutant has relatively lower usage for that particular group of start sites and a positive value indicates a relatively higher usage for that particular group of start sites. Values shown are the average of at least four independent experiments +/− standard deviation. B. Primer extension analysis of RNA 5′-ends at *HIS4* and *PMA1* in Pol II TL mutants. C. Primer extension analysis of RNA 5′-ends at *GAL1* in Pol II TL mutants. Cells are grown in medium lacking raffinose and then *GAL1* is induced by addition of galactose to 2% for two hours. Sequence ladder at left is derived from the same primer used for primer extension. Numbers indicate positions of putative start sites in relation to the *GAL1* ATG (A is +1).

A number of Pol II subunit and general transcription factor (GTF) mutants have been found to alter start site selection in *S. cerevisiae* [73], [75]–[84], but it is unclear how Pol II activity might relate to these start site defects. For example, deletion of the Rpb9 subunit of Pol II confers many similar activity defects as Pol II TL GOF substitutions such as increased rate of elongation and misincorporation [85]–[87] and is defective for start site switching at *IMD2* in response to MPA [58]. Additionally, *rpb9*Δ has long been known to exhibit upstream shifts in start site selection [77], [79], [84], while many mutations throughout Pol II can cause downstream shifts in start site selection (references above). Models for Pol II residue function in start site selection have previously focused on physical or functional interactions between these residues and GTFs. We propose that the Pol II TL primarily or exclusively functions in controlling the Pol II nucleotide addition cycle (substrate selection, catalysis and translocation), which, for this work, we define as Pol II activity. Therefore we used our TL mutants to interrogate the relationship between Pol II biochemical activity and start site selection *in vivo*, following our observations of downstream start site usage at *IMD2* in TL LOF H1085Y and apparent upstream shifts in TL GOF E1103G and G1097D mutants. We examined start site selection at a number of other genes by primer extension analysis (Figure 6).

We found that TL LOF H1085Y caused downstream shift in start site distribution at *ADH1*, similar to reported defects of a number of other Pol II mutants and GTF mutants (Figure 6A). In contrast, TL GOF E1103G and G1097D substitutions confer upstream defects similar to those that occur in *rpb9*Δ (references above), a number of TFIIF mutants [78], [80]–[83], and novel insertion mutants within TFIIB [88]. As with other *in vivo* and *in vitro* phenotypes, combination of H1085Y and E1103G leads to mutual suppression, extending the correlation between observed phenotypes and *in vitro* activity. For transcripts from *HIS4* and *PMA1*, genes known to be sensitive to start site alterations in mutant strains [73], [79], we also observed upstream shifts in E1103G and G1097D-bearing strains (Figure 6B). E1103G-dependent start site changes were suppressed by H1085Y for these genes as well. It is not obvious how the alteration of TL function would impact start site selection so strongly, as we expect TL function to be downstream of initiation events important for start site selection, such as pre-initiation complex formation and promoter melting. The strong upstream starts observed for GOF mutants led us to predict that these positions should already be accessible to Pol II in WT cells, because TL function in controlling Pol II activity should be downstream of promoter melting. Using permanganate footprinting to identify regions of single-stranded DNA in the *GAL1* and *GAL10* promoters, Giardina and Lis observed strong permanganate reactivity at greater than 60 basepairs (bp) upstream of the major transcriptional start sites, indicating promoter melting far upstream [89]. We reasoned that *GAL1* should show far upstream start site shifts in TL GOF mutants within this melted region though it does not normally support productive start sites. Indeed, we observed new start site usage more than 40 bp upstream of the major *GAL1* start sites in TL GOF mutants, and a slight shift in starts sites downstream in TL LOF H1085Y (Figure 6C)(Figure S7).

## Discussion

The contributions of TL residues to the Pol II nucleotide addition cycle (“activity” for the purposes of this discussion) are critical for transcription *in vitro* and *in vivo*. The catalytic contribution of the TL has been proposed to function through minimization of NTP dynamics within the Pol II active site, which in turn could increase the probability of catalysis [90]. Our biochemical experiments coupled with extensive genetic analyses provide insight into the function of TL residues. We calculated an approximate 6-fold suppression effect on elongation rate conferred by the E1103G GOF substitution when it was combined with LOF substitutions. We used this fold enhancement to infer the putative defects of inviable TL LOF substitution mutants for which we were unable to measure elongation rate directly. Assuming an approximate 6-fold increase in rate contributed by E1103G to the values of E1103G-suppressed inviable substitution elongation rates, we infer 7–30 fold defects for N1082A, H1085A and Q1078A single substitutions (Figure 4B). Examination of analogous substitutions in *E. coli* or *T. thermophilus* RNAPs has revealed large differences in the contribution of these residues to catalysis with NTP substrates (4 and 6 fold for *Eco* R933A*^Sce^* ^N1082A^ and H936A*^Sce^* ^H1085A^, respectively [7] vs. 50 and 100 fold for *Tth* R1239A*^Sce^* ^N1082A^ and H1242A*^Sce^* ^H1085A^, respectively [5]). Our results indicate that *S. cerevisiae* Pol II TL residues have contributions intermediate to TL residues from different bacteria and suggest that contributions of TL residues to activity cover a range of values in homologous enzymes.

Our data also support a functional relationship between regions adjacent to TL hinges, where the TL has been observed to deviate from its fully folded, NTP-bound conformation in crystal structures. These regions are the location of most of the strong Spt^−^ and MPA^s^ TL substitutions, as well as the strongest GOF alleles tested. These results underscore the idea that the TL is finely balanced between functional states or conformations, and that the hinges are sensitive to changes that in some cases can promote Pol II activity. These changes likely occur at the expense of fidelity, as has been shown for E1103G [2], [9]. Alteration of distal TL residues (*e.g.* E1103, G1097D) conferred broad suppression on a number of TL NIR substitutions. Thus, it appears that loss of NIR function can be compensated by alteration in trigger loop dynamics. Importantly, functions of TL NIR residues are not completely bypassed, as activities of tested double mutants are intermediate between the single mutants, and not all NIR mutants are suppressible (Table 1, Text S1).

**Figure 7:**
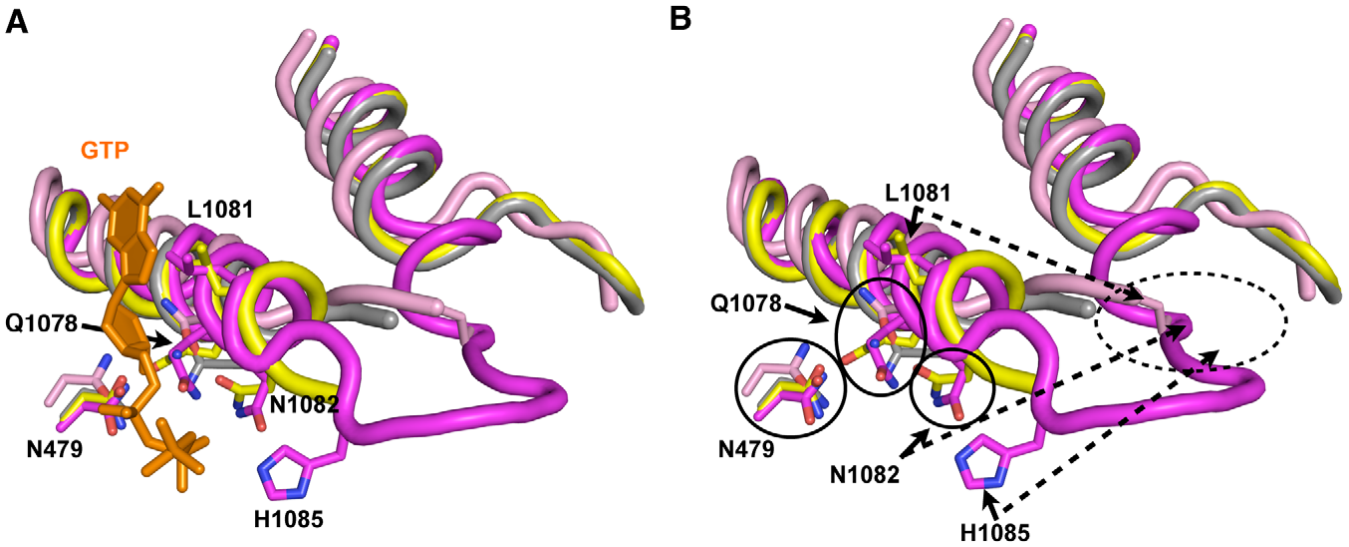
Model for stepwise function of TL residues in contributing to TL folding and function. A. Structural cartoon showing overlay of TLs and Rpb1 N479 from a number of Pol II elongation complex/initial transcribing complex crystal structures with TLs in different states of folding/NTP interaction. Matched GTP nucleotide (orange) and TL and N479 (magenta) are from PDP 2E2H [1], which shows TL in a closed conformation. Partially folded TL and N479 (yellow) from PDB 4A3F [13], which contains a matched NTP analog (not shown) and short RNA (not shown). Related structure to PDB 4A3F from PDB 4A3D [13], but without matched NTP analog is shown in gray. Partially folded TL from PDB 1R9T, containing a mismatched NTP (not shown) is shown in pink. B. Same figure as in (A) but with GTP omitted. Certain residues are relatively closely positioned in all structures overlaid (N479, Q1078), while other residues are observed stabilized in partially folded structures directly adjacent to the active site (L1081, N1082). H1085 is observed positioned adjacent to the active site only upon complete folding/stabilization of the TL. Dashed oval indicates likely location of TL NIR residues when in out conformation where the TL is either mobile or unfolded. This figure was created with Pymol [111].

Functional distinctions between TL residues and genetic epistasis between some residue combinations have been hinted at in the case of residues analogous to Q1078 and N1082 in *T. thermophilus* experiments [5](Text S1), but our combinatorial genetic analyses reveal this more clearly (Figure 4). Examination of different Pol II crystal structures shows the TL in a number of states between partially and stably folded in the presence or absence of matched NTP or NTP analog [1], [13], [67](Figure 7). These structures suggest a TL folding pathway wherein N-terminal TL residues that are proximal to the N-terminal TL hinge (primarily Q1078), along with other active site residues (for example, Rpb1 R446 and N479), are positioned essentially at all times for NTP interaction. Biochemical experiments indicate that these residues function in ribose 2′- and 3′-hydroxyl (OH) recognition, with the 2′-OH of course critical for *selection* of NTPs over 2′-dNTPs [1], [5], [91]. Upon matched NTP basepairing with the DNA template, NTP interactions with R446, N479 and Q1078 may stabilize or begin to promote TL-folding in an N-terminal to C-terminal fashion, where L1081 and N1082 next come into position via N-terminal TL hinge movement. Supporting such a step, in some Pol II crystal structures, these residues are observed adjacent to a matched NTP basepairing with the template, but without complete folding or stabilization of the TL (Figure 7). Our genetic analyses suggest that Q1078 and N1082 (along with N479) are functionally interdependent (see also Text S1). Following initial hinge movement and TL folding, H1085 and the rest of the TL loop tip must fold into the closed conformation, and genetic analyses of H1085 substitutions suggest this step is distinguishable from functions of other TL NIR residues. It is important to note that we have not been able to evaluate contributions of residues L1081 and R446 in our genetic system because all tested substitutions in these residues were inviable and these substitutions were generally not suppressed by E1103G (Table 1, Table S1, Text S1).

### The Relationship between MPA Sensitivity and Pol II Elongation Defects

*In vivo* it has been observed that some Pol II mutants constitutively express *IMD2* in the absence of drug-induced stimulation [18], [47]. It is unclear whether this constitutive expression represents a mutant-induced alteration in Pol II start site specificity or a generic reduction of Pol II activity that mimics a drug-induced nucleotide depleted state, which itself leads to altered start site selection. We show here that an MPA-resistant LOF TL allele caused constitutive *IMD2* expression, and in other work we found that this expression is likely a generic hallmark for reduced Pol II activity (Braberg et al, in preparation), and not necessarily due to a specific defect in initiation as previously proposed [57]. These results support published models that *IMD2* regulation is directly responsive to reduction in nucleotide levels as reduction or increase in Pol II catalytic rates have opposing effects on the ability of *IMD2* to be induced. Our observations that reduced Pol II activity does not correlate with sensitivity to MPA suggest that generic Pol II catalytic defects do not confer sensitivity to reduced nucleotide levels. Given the fact that LOF TL mutants are highly likely to confer elongation defects *in vivo*, the absence of MPA-sensitivity raises the question of whether reduction of GTP levels sensitizes cells to generic elongation defects caused by *any* transcription factor proposed to be acting through Pol II elongation. MPA sensitivity of any transcription factor mutant should not be used to infer presence or absence of an elongation defect because actual nucleotide levels in different mutant strains may vastly differ due to *IMD2* transcriptional effects.

### Model for Start Site Selection in Budding Yeast

**Figure 8:**
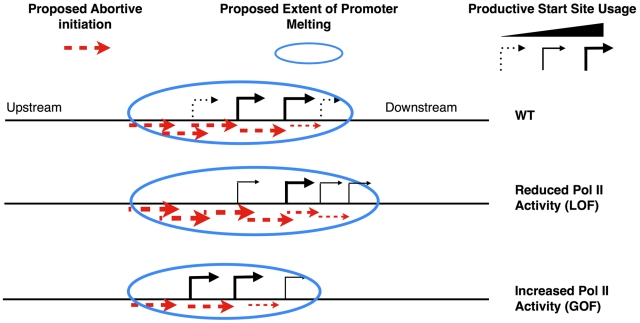
Model for transcription-assisted start site scanning through abortive initiation. Cartoon showing predicted start site distribution for an *ADH1*-like gene in WT cells (top) or alteration in distribution in the presence of LOF (middle) or GOF (bottom) Pol II TL mutants.

The observation that TL mutants show polar effects on start site selection *in vivo* correlating with *in vitro* catalytic defects suggests a model for how Pol II activity may directly influence initiation. Start site selection in *S. cerevisiae* is not restricted to a short window a defined distance from the TATA box, but instead can take place over a longer region downstream [92]. It has been proposed that Pol II scans for sequences permissive for productive elongation [89]. We propose that our mutants primarily affect the nucleotide addition cycle and not some other step in initiation, therefore we expect promoter melting is already occurring in regions where start sites are not normally utilized in WT cells, and that scanning occurs subsequent to template melting, as has been proposed for *GAL* genes [89]. *Continuous* RNA synthesis during the scanning process does not appear to be required for scanning, as experiments show that chain-terminating nucleotides do not abrogate scanning *in vitro* [93], [94]. In light of these results, models for start site scanning in yeast that do not invoke transcription have been proposed (*i.e.* [95], also reviewed in [96]). We show that substitutions within the Pol II TL that have increased activity *in vitro* shift start sites upstream at *ADH1*, *HIS3*, *PMA1* and *GAL1*, and those with reduced *in vitro* activity shift start sites downstream at *ADH1* and *GAL1* (Figure 6 and Figure S8). To explain these data, we invoke a new transcription-assisted model for start site scanning (Figure 8). Inefficiently or apparently unused start sites may allow undetected *abortive* but not productive initiation. Such abortive transcription may support Pol II downstream translocation, providing an explanation how Pol II catalytic mutants alter start site selection in a polar fashion, which is indicative of altered scanning as described in the flux model proposed by the Brow lab [97]. In the flux model, Pol II scanning from upstream to downstream will utilize starts in a polar fashion, and a decrease in usage of an upstream start will lead to increased usage of a downstream start. In our model, upstream shifts in start sites are due to increased Pol II catalytic activity, which in turn increases the probability of normally abortive initiation events becoming productive at the expense of downstream events. Conversely, downstream shifts in start sites are due to decreased Pol II activity, which increases the probability that abortive initiation will occur at upstream positions at the expense of productive initiation from those positions. Competition between abortive and productive initiation may occur at key transitions for early elongation complex stability [98], or by modulating stability of RNA:DNA hybrid in early transcription complexes as suggested by recent structural studies from the Kornberg lab [99] and the Cramer lab [13]. These new structures demonstrated a role for a matched NTP substrate in the Pol II active site in stabilization of short nascent RNAs. As GOF Pol II TL alleles have been implicated in extending the duration of substrate-bound complexes [9], these GOF alleles may also stabilize early elongation complexes in addition to increasing elongation rate. Any increase in the probability of next nucleotide incorporation at the expense of transcript release would lead to a net *increase* in productive elongation and a net *decrease* in downstream scanning by our model.

Previously described start site-altering mutants in Pol II general factors and other Pol II subunits have activities that are entirely consistent with this model. *rpb9*Δ shows upstream shifts in start sites at *ADH1* (and other genes) [77], [79], inability to shift to downstream *IMD2* starts upon MPA treatment, and causes Pol II to be superactive *in vitro* [85], similarly to superactive TL mutants E1103G and G1097D. An allele of the TFIIF subunit-encoding *TFG1* gene causes upstream start site shifts *in vivo* while increasing efficiency of Pol II initiation in an abortive initiation assay, consistent with this allele conferring an increase in Pol II activity [94]. This increase in Pol II activity may be due to a gain of function in TFIIF or a loss of a negative function, under our activity-dependent framework for interpreting start site defects. Consistent with the latter, a recent *in vitro* study shows that TFIIF can inhibit initiation at specific template positions on a modified *HIS4* template [100]. On some of the templates used in this study, TFIIF inhibits transcription initiation from upstream positions, consistent with polarity of most start site shifting mutants of TFIIF subunits *in vivo*. A number of *sua7* (TFIIB) alleles show downstream shifts in start site selection [76], [101], [102], and in the case of the highly studied E62K allele, this mutant causes a decrease in Pol II transcription efficiency *in vitro* [103]. We provide a framework for interpreting the relationship between start site selection changes and the nature of alteration of the initiation process. Increasing or decreasing efficiency of productive initiation from particular nucleotide positions may relate to upstream or downstream shifts in start site selection, which appear to correlate with increases or decreases in Pol II activity, respectively. While start site defects strongly correlate with Pol II activity, their contribution to the growth defects of Pol II TL mutants remains to be determined.

## Materials and Methods

### Genetic Analyses

To examine mutant polymerase alleles encoding substitutions in the TL and elsewhere in Rpb1, we perform a plasmid shuffle with *rpb1 CEN LEU2* plasmids by assaying ability of *rpb1*Δ cells to grow without an *RPB1 CEN URA3* plasmid. This is accomplished by treatment of cells with the drug 5-fluoroorotic acid (5-FOA, Gold Biotechnology), which is toxic to Ura3^+^ cells [104], thus selecting cells containing solely an *rpb1 CEN LEU2* plasmid so we might assess the ability of mutant plasmids to complement the essential function of *RPB1*. During the course of our experiments, we identified a previously unreported polymorphism in our *RPB1* plasmid encoding substitution of isoleucine at position 69 for the threonine reported for this position in the Yeast Genome Database (T69I). This substitution was tracked as far back as the pRP112 subclone derived from an original *RPO21* genomic clone from the Young lab [105]. Our analyses indicate that this substitution is phenotypically inert (Figure S8), yet all genetic data shown are for plasmids with the Ile69 corrected to threonine (I69T).

### Yeast Strains, Plasmids, and Media

Yeast media are prepared in standard fashion as described in [106] with minor alterations. Yeast extract (1%)(BD), peptone (2%)(BD), dextrose (2%)(“YPD”) solid medium (2% bacto-agar, BD) is supplemented with adenine (0.15 mM final) and tryptophan (0.4 mM final)(Sigma-Aldrich). YP plates with alternate carbon sources such as raffinose (2%, USB) or raffinose (2%) plus galactose (1%, Sigma-Aldrich) also contain antimycin A (1 µg/ml, Sigma-Aldrich). Minimal media plates are synthetic complete (“SC”/“Hopkins mix”) with amino-acids dropped out as appropriate as described in [106] with minor alterations: per standard batch formulation Adenine Hemisulfate was 2 g, Uracil was 2 g, myo-inositol was 0.1 g, p-Aminobenzoic acid (PABA) was 0.2 g. For studies with mycophenolic acid (MPA, Sigma-Aldrich), MPA was added to minimal SC-Leucine medium at 20 µg/ml from a 10 mg/ml stock in ethanol. Construction of mutants is described in Text S1. Yeast strain genotypes, numbers and plasmid descriptions are found in Tables S1 and S2.

### Pol II Purification and *In Vitro* Transcription Assays

Pol II enzymes were purified from yeast strains expressing mutant *rpb1* genes from a low copy plasmid from the endogenous *RPO21* promoter via a tandem-affinity tag (TAP) on Rpb3, in a procedure derived from [107] and as described in [2]. All mutant enzymes are Rpb1 I69 variants, except H1085Q/E1103G, F1086S/E1103G and Q1078A/E1103G, which are T69 variants. Transcription reactions of Pol II elongation complexes formed on nucleic acid scaffolds follow the approach described by Komissarova *et al* [108], and are performed as in [2] with the following modifications: for some experiments, amounts of all nucleic acids were reduced to 1/10^th^ the amount stated in [2] (to 30 pmol from 300 pmol, *etc.*)(Text S1). Reactions were separated on 13.5% acrylamide-bisacrylamide gels (19∶1 ratio) containing 1× TBE and 7 M urea and quantitated as previously described [2].

### Primer Extension and Northern Blotting Analysis

Total yeast RNA was purified as described [109]. Primer extension analysis was performed exactly as described (http://labs.fhcrc.org/hahn/Methods/mol_bio_meth/primer_ext.html) [110] with the following modifications. Total RNA used was 30 µg, and in the case of more dilute RNA samples, volumes of reactions were increased by 50% to accommodate greater volume of sample. Reverse transcriptase (RT) was M-MLV RT from either Life Technologies or Fermentas. RT synthesis reactions were supplemented with 1 µl RNAse Inhibitor (Fermentas). Extension products were separated on either 7% or 10% acrylamide-bisacrylamide gels (19∶1 ratio) containing 1× TBE and 7 M Urea. Northern blotting was performed essentially as described in manual for GeneScreen hybridization membranes (Perkin-Elmer) with the following modifications. RNA samples (20 µg) were prepared in NorthernMax loading buffer (Ambion/AB). Prehybridization solution did not contain SSPE or SSC buffers, but contained 5× Denhardt's solution, 50 mM Tris-HCl pH 7.5, 1 M NaCl, 0.1% sodium pyrophosphate, 0.1% SDS instead of 1%, 10% Dextran Sulfate, 50% formamide, and 500 µg/ml sheared/denatured salmon sperm DNA. Probes for northern blots were radiolabeled using α-^32^P-dATP by random priming using the Decaprime II kit (Ambion) according to manufacturer's instructions. Blots were washed at twice for 10 minutes each wash at 42°C with 2× SSC, 0.5% SDS, then twice at 67°C with 5× SSC, 0.5% SDS for 30 minutes each wash, then twice in 0.2× SSC for 30 minutes each wash at room temperature. Primer extension gels and northern blots were visualized by phosphorimaging (GE Healthcare or Bio-Rad) and quantified using ImageQuant 5.0 (GE) or Quantity One (Bio-Rad) software, with data exported to Microsoft Excel for management. Oligo sequences for site-directed mutagenesis, primer extension analysis, amplification of DNA for Northern blotting and *in vitro* transcription are available upon request.

## Supporting Information

Figure S1 *In vivo* transcriptional phenotypes utilized in this study. A. The Spt^−^ phenotype utilized in this work relates to suppression of a Ty1 delta (∂) element insertion into the 5′ end of the *LYS2* coding region creating the *lys2-128∂* allele [59]. This insertion renders WT cells Lys^−^ (top) as they are only able to express a short non-functional transcript (black arrow), while mutation of a number of factors allows transcription of *LYS2* from a cryptic promoter (red arrow, bottom), most likely somewhere within the ∂ insertion, allowing the cells to become Lys^+^. B. The galactose toxicity phenotype utilized here relates to transcriptional interference between *GAL10* transcription and *GAL7* transcription caused by compromise of *GAL10* 3′-end formation through deletion of the major polyadenylation signal (*gal10*Δ*56*) and subsequent interference with *GAL7* initiation (black arrows) [60], [61], [112]. Decrease in *GAL7* transcription allows the buildup of a toxic metabolite normally metabolized by Gal7p, thus presence of galactose in the medium becomes toxic under conditions where other *GAL* genes are expressed. Mutations in a number of factors that enhance *GAL10* 3′-end formation, enhance termination downstream of *GAL10*, or increase *GAL7* transcription can suppress this toxicity (red arrows).
(TIF)

Figure S2 Genetic analyses of Pol II TL single substitution mutants combined with E1103G or G1097D substitutions distinguish between different classes of Pol II mutant and show extensive suppressive relationships. A. 10-fold serial dilutions of saturated cultures for a number of Pol II TL substitutions combined with E1103G plated on different media as in Figure 1, with single mutant panels from Figure 1 shown for comparison purposes. B. 10-fold serial dilutions of saturated cultures for a number of Pol II TL substitutions combined with G1097D are plated on different media as in Figure 1.
(TIF)

Figure S3 Additional Pol II TL single substitution and multiple substitution mutants. 10-fold serial dilutions of saturated cultures of Pol II TL mutant strains plated on different media. Also note that the multiply substituted Q1078S/N1082A/H1085Y/E1103G mutant is viable but confers a strong growth defect. When combined with an A1076V substitution, previously identified as *sit1-8G* (as a double mutant with *rpb1*-N479Y conferring a phenotype reminiscent of the Spt^−^ phenotype) [22], we observed that A1076V suppressed growth defects of Q1078S/N1082A/H1085Y/E1103G, and that it also confers viability to Q1078A/N1082A/H1085Y/E1103G.
(TIF)

Figure S4 Quantification of elongation rates in Pol II mutants. Run-off transcription as a fraction of total transcription is determined and plotted versus reaction time for specified Pol II mutants. Multiple lines per graph indicate data from time courses generated from separate individual reactions. A. WT I69. B. WT T69. C. N1082S. D. H1085Q. E. L1101S. F. E1103G. G. F1086S/E1103G. H. H1085Q/E1103G. I. H1085Y/E1103G. J. N1082A/E1103G. K. H1085A/E1103G. L. Q1078A/E1103G. M. G1097D with standard NTP concentrations. N. G1097D with high NTP concentrations (MgCl_2_ raised to 10 mM from 5 mM).
(TIF)

Figure S5 Quantification of elongation rates in Pol II mutants. Rates determined from Figure S4 for each NTP concentration are plotted versus NTP concentration and curve fitted by non-linear regression. A. WT Pol II enzymes. B. Single mutant Pol II enzymes. C. Double mutant Pol II enzymes. D. G1097D with standard NTP concentrations. E. G1097D with high NTP concentrations (MgCl_2_ raised to 10 mM from 5 mM).
(TIF)

Figure S6 Western blotting for Rpb1 and Rpb3-TAP from Pol II variants. A. Western blotting for Rpb1 expression levels from WT and *rpb1* mutant yeast strains using an antibody to the N-terminus of Rpb1 (Text S1). Because our strains contain Rpb3-TAP, the protein A tag on Rpb3-TAP was also recognized by either the primary or secondary antibodies used. Blotting for Pgk1 was used to confirm equal loading of lanes (bottom panels). Blots shown are representative of three independent experiments. B. Quantification of Rpb1/Pgk1 ratio from three independent experiments. Error bars indicate +/− standard deviation of the mean. The anti-Rpb1 antibody has a high background, and subtraction of this background caused standard deviations to generally increase, so both background subtracted and unsubtracted quantifications are shown.
(TIF)

Figure S7 Quantification of start site distribution at *GAL1* in Pol II mutants. Start site usage at *GAL1* as determined by primer extension with radiolabeled oligo was divided into bins based on radioactive signal at positions of start sites relative to *GAL1* ATG sequence (A is +1) and normalized to total signal per lane. WT start site fractions were subtracted from mutant start site fractions for particular regions to determine the relative alteration in start site distribution in Pol II mutant strains. A negative value indicates that the mutant has relatively lower usage for that particular group of start sites and a positive value indicates a relatively higher usage for that particular group of start sites. Values shown are the average of three independent experiments +/− standard deviation.
(TIF)

Figure S8 No effect of Rpb1 I69 substitution on *in vivo* phenotypes. 10-fold serial dilutions of yeast strains expressing T69 or I69 variants of Rpb1 as the sole source of Rpb1 were spotted onto various media to determine phenotypes. Results indicate that I69 is phenotypically inert.
(TIF)

Table S1 *rpb1* mutant plasmids used in genetic analyses of the Pol II TL.
(DOCX)

Table S2 Strain genotypes for yeast used in this study.
(DOCX)

Text S1 Description of plasmids, yeast strains, supporting discussion of genetic analyses of Pol II TL residues, and supporting methods.
(DOCX)
