# Supplementary figures and images for: Dissection of Pol II Trigger Loop Function and Pol II Activity–Dependent Control of Start Site Selection In Vivo
Source: PLoS Genet. 2012 Apr 12;8(4):e1002627. doi: 10.1371/journal.pgen.1002627 (PMC3325174; doi:10.1371/journal.pgen.1002627)

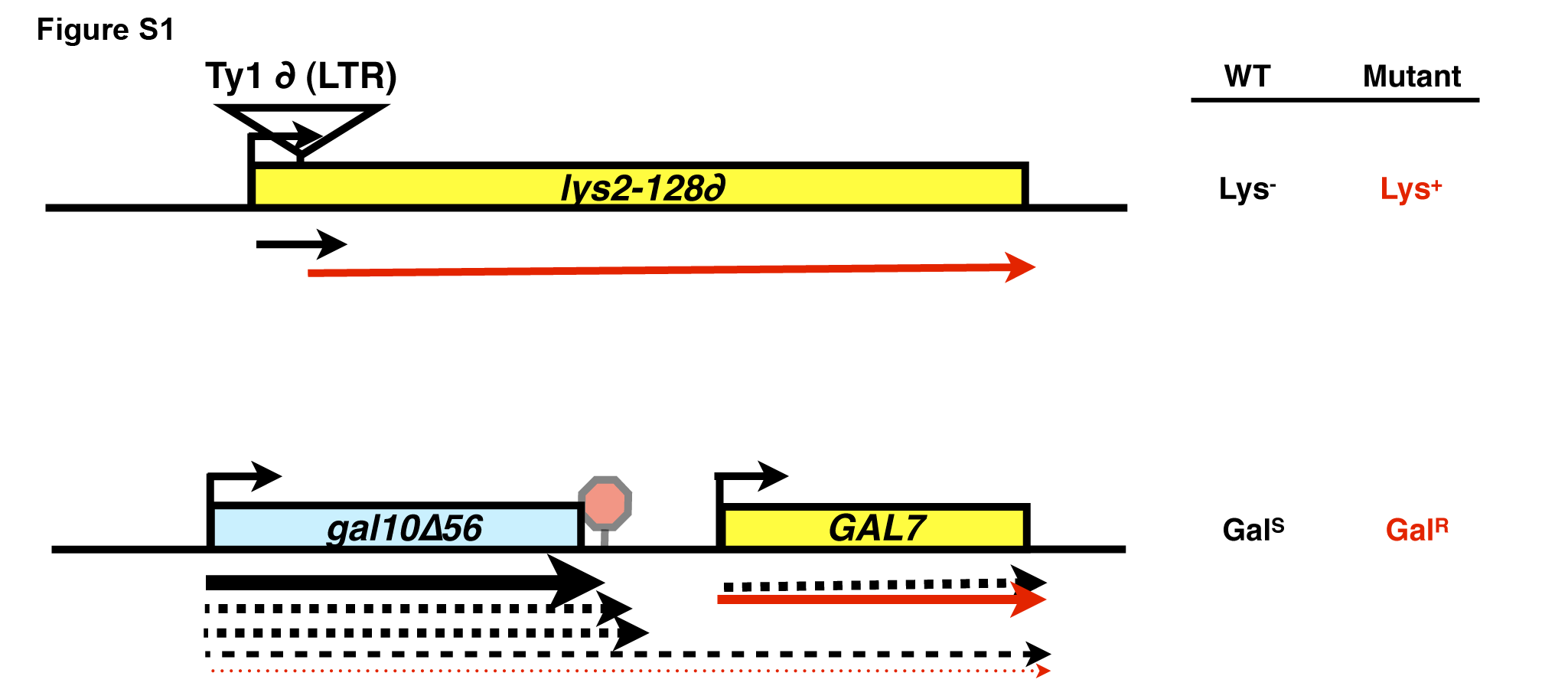

Supplement: Figure S1 — In vivo transcriptional phenotypes utilized in this study. A. The Spt− phenotype utilized in this work relates to suppression of a Ty1 delta (∂) element insertion into the 5′ end of the LYS2 coding region creating the lys2-128∂ allele [59]. This insertion renders WT cells Lys− (top) as they are only able to express a short non-functional transcript (black arrow), while mutation of a number of factors allows transcription of LYS2 from a cryptic promoter (red arrow, bottom), most likely somewhere within the ∂ insertion, allowing the cells to become Lys+. B. The galactose toxicity phenotype utilized here relates to transcriptional interference between GAL10 transcription and GAL7 transcription caused by compromise of GAL10 3′-end formation through deletion of the major polyadenylation signal (gal10Δ56) and subsequent interference with GAL7 initiation (black arrows) [60], [61], [112]. Decrease in GAL7 transcription allows the buildup of a toxic metabolite normally metabolized by Gal7p, thus presence of galactose in the medium becomes toxic under conditions where other GAL genes are expressed. Mutations in a number of factors that enhance GAL10 3′-end formation, enhance termination downstream of GAL10, or increase GAL7 transcription can suppress this toxicity (red arrows). (TIF) [file pgen.1002627.s001.tif]

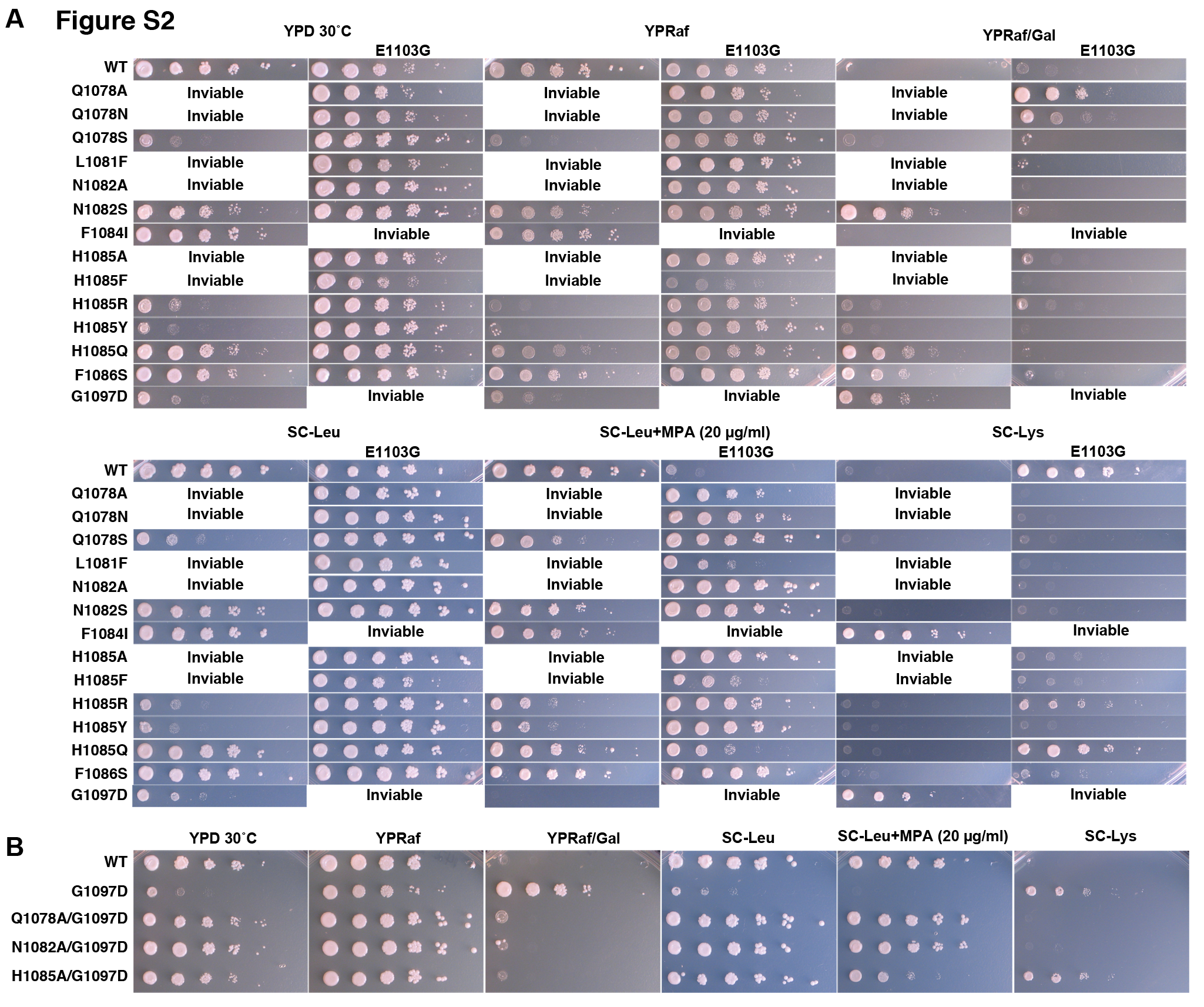

Supplement: Figure S2 — Genetic analyses of Pol II TL single substitution mutants combined with E1103G or G1097D substitutions distinguish between different classes of Pol II mutant and show extensive suppressive relationships. A. 10-fold serial dilutions of saturated cultures for a number of Pol II TL substitutions combined with E1103G plated on different media as in Figure 1, with single mutant panels from Figure 1 shown for comparison purposes. B. 10-fold serial dilutions of saturated cultures for a number of Pol II TL substitutions combined with G1097D are plated on different media as in Figure 1. (TIF) [file pgen.1002627.s002.tif]

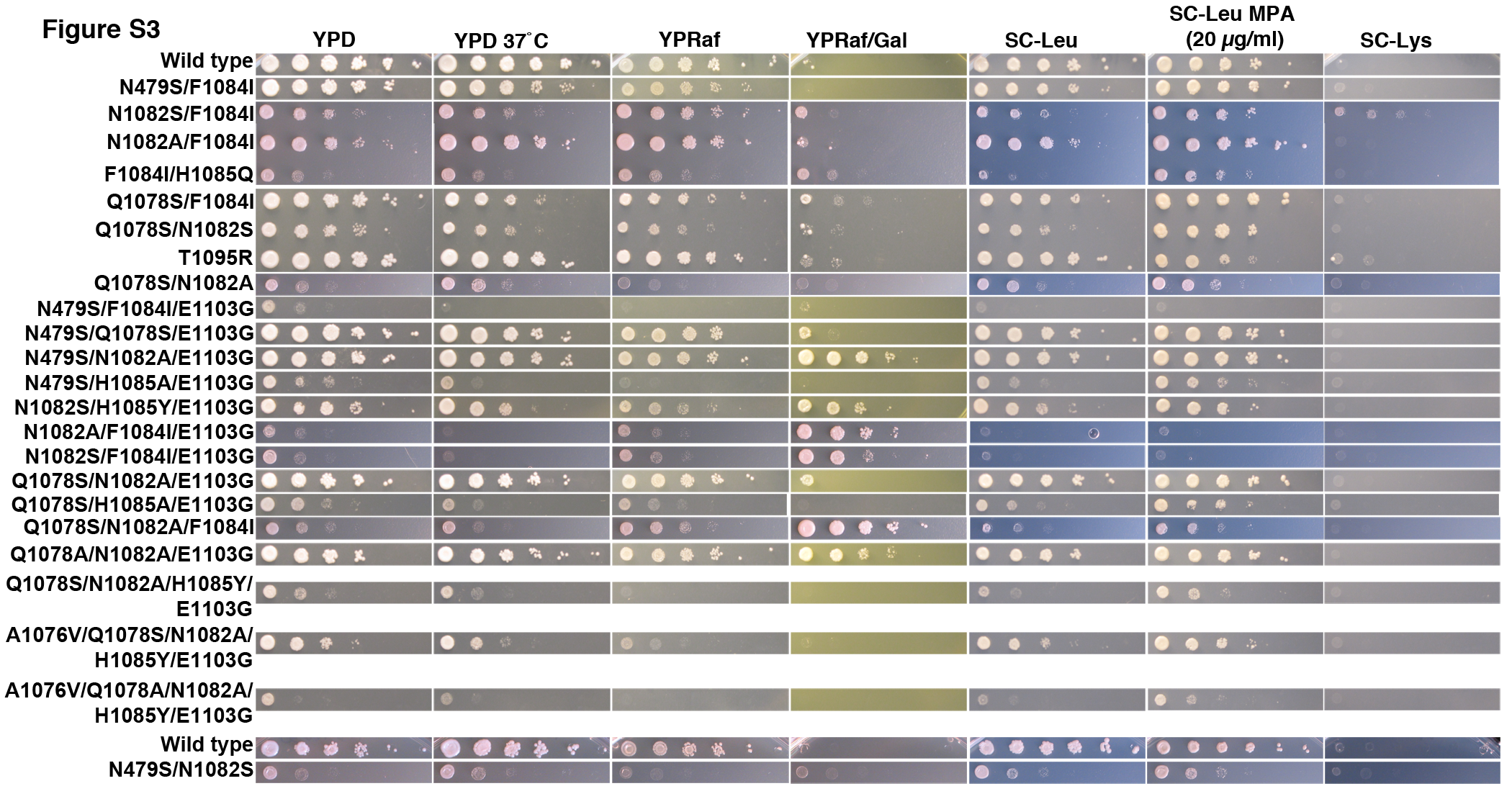

Supplement: Figure S3 — Additional Pol II TL single substitution and multiple substitution mutants. 10-fold serial dilutions of saturated cultures of Pol II TL mutant strains plated on different media. Also note that the multiply substituted Q1078S/N1082A/H1085Y/E1103G mutant is viable but confers a strong growth defect. When combined with an A1076V substitution, previously identified as sit1-8G (as a double mutant with rpb1-N479Y conferring a phenotype reminiscent of the Spt− phenotype) [22], we observed that A1076V suppressed growth defects of Q1078S/N1082A/H1085Y/E1103G, and that it also confers viability to Q1078A/N1082A/H1085Y/E1103G. (TIF) [file pgen.1002627.s003.tif]

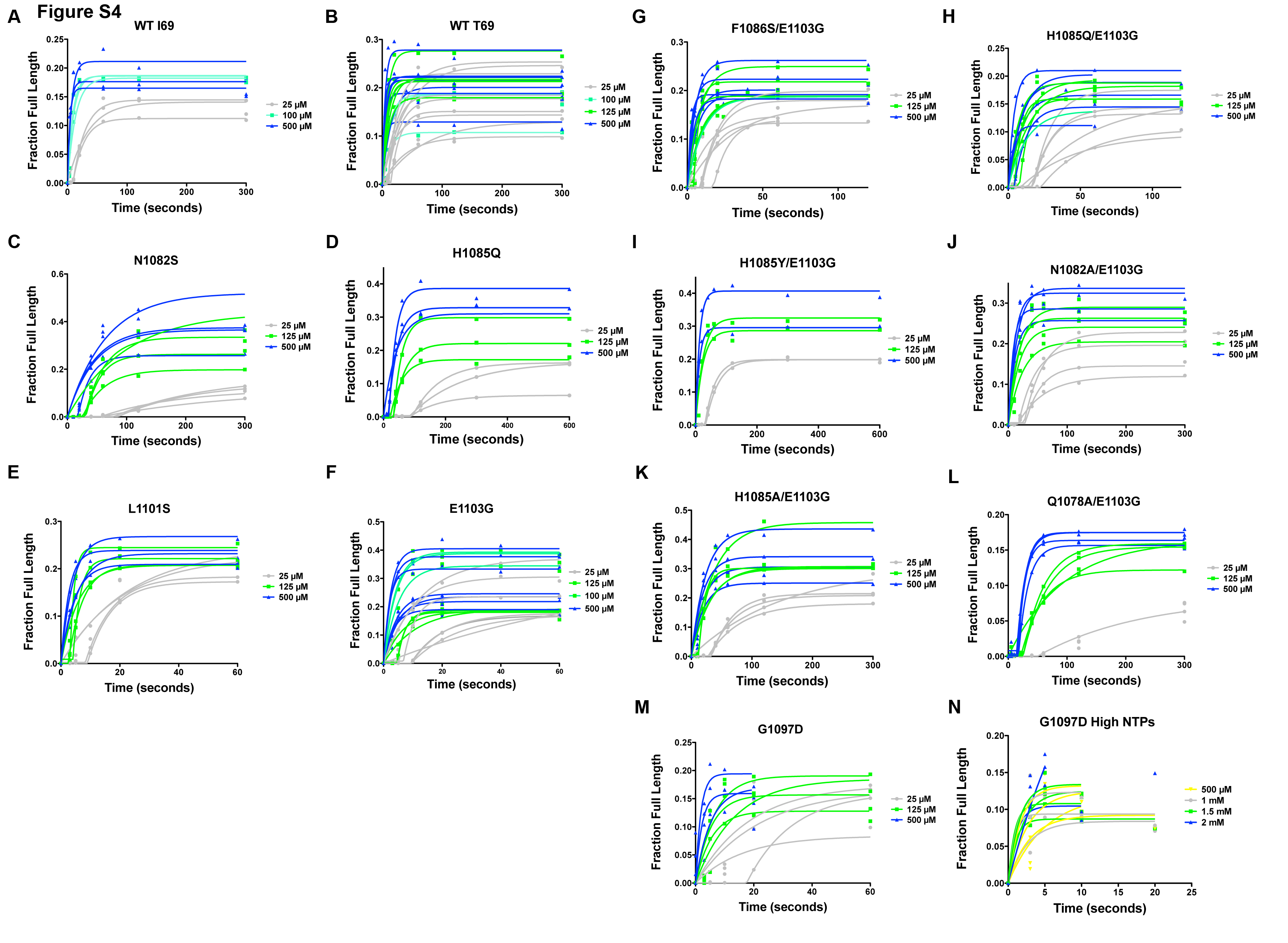

Supplement: Figure S4 — Quantification of elongation rates in Pol II mutants. Run-off transcription as a fraction of total transcription is determined and plotted versus reaction time for specified Pol II mutants. Multiple lines per graph indicate data from time courses generated from separate individual reactions. A. WT I69. B. WT T69. C. N1082S. D. H1085Q. E. L1101S. F. E1103G. G. F1086S/E1103G. H. H1085Q/E1103G. I. H1085Y/E1103G. J. N1082A/E1103G. K. H1085A/E1103G. L. Q1078A/E1103G. M. G1097D with standard NTP concentrations. N. G1097D with high NTP concentrations (MgCl2 raised to 10 mM from 5 mM). (TIF) [file pgen.1002627.s004.tif]

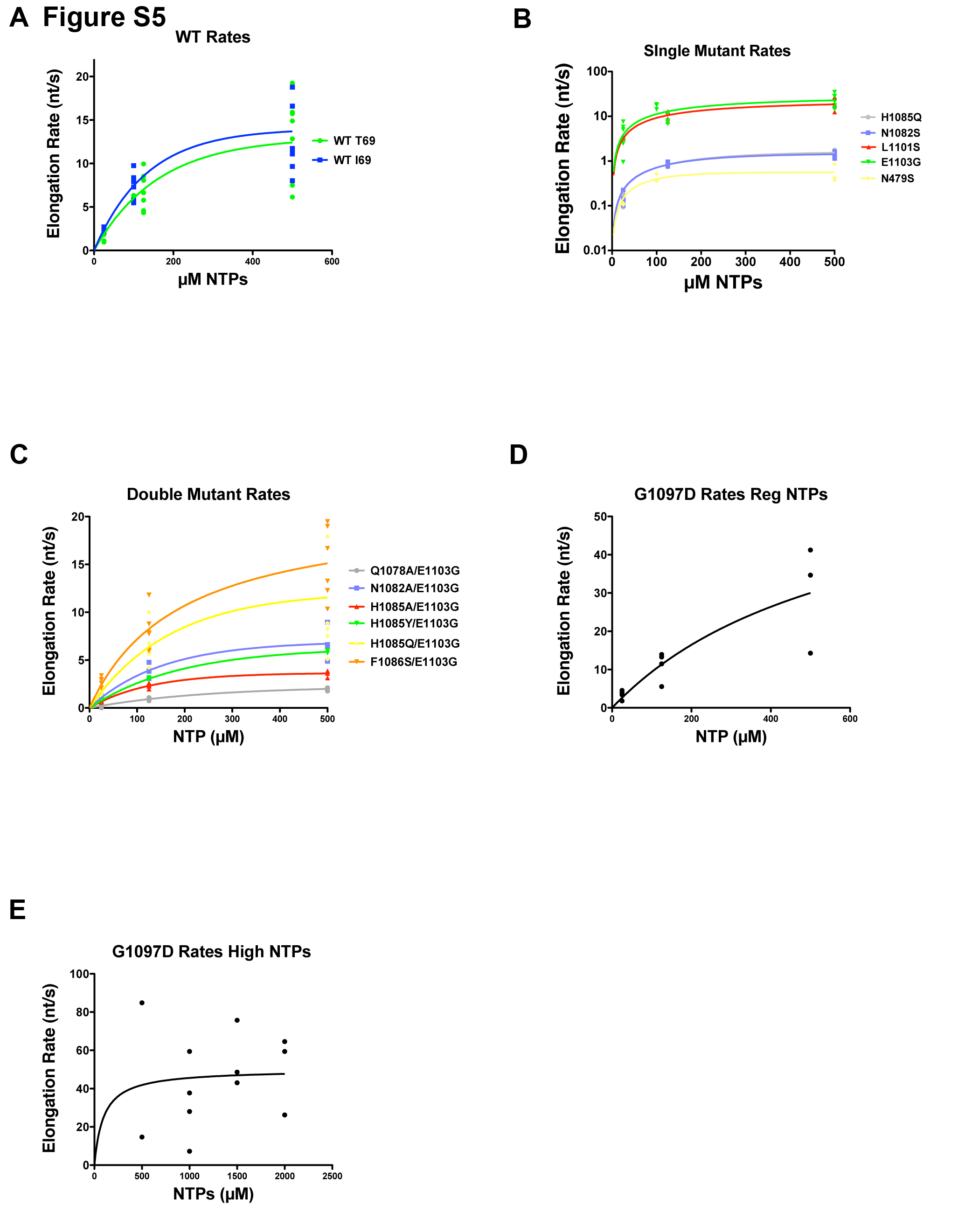

Supplement: Figure S5 — Quantification of elongation rates in Pol II mutants. Rates determined from Figure S4 for each NTP concentration are plotted versus NTP concentration and curve fitted by non-linear regression. A. WT Pol II enzymes. B. Single mutant Pol II enzymes. C. Double mutant Pol II enzymes. D. G1097D with standard NTP concentrations. E. G1097D with high NTP concentrations (MgCl2 raised to 10 mM from 5 mM). (TIF) [file pgen.1002627.s005.tif]

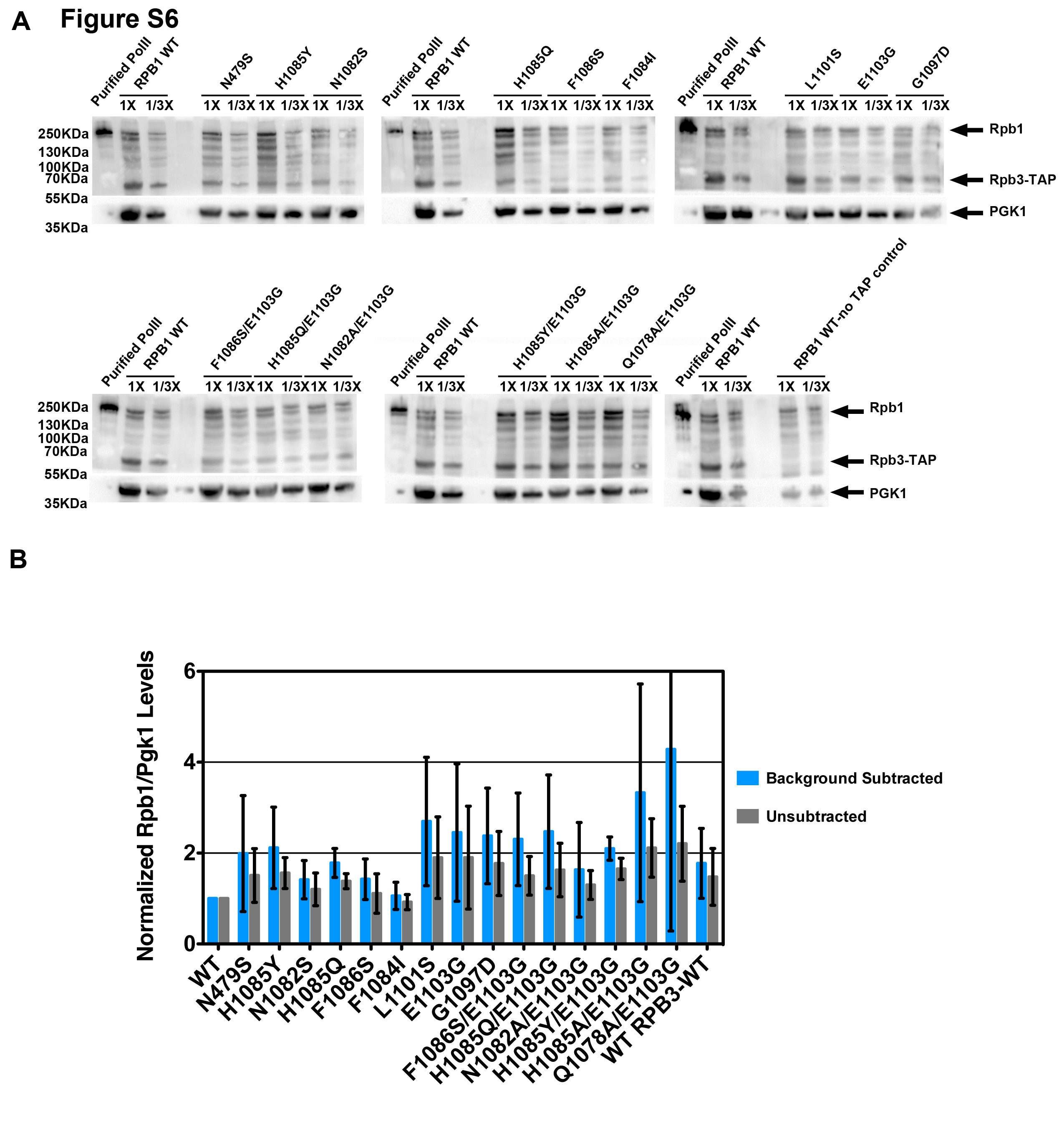

Supplement: Figure S6 — Western blotting for Rpb1 and Rpb3-TAP from Pol II variants. A. Western blotting for Rpb1 expression levels from WT and rpb1 mutant yeast strains using an antibody to the N-terminus of Rpb1 (Text S1). Because our strains contain Rpb3-TAP, the protein A tag on Rpb3-TAP was also recognized by either the primary or secondary antibodies used. Blotting for Pgk1 was used to confirm equal loading of lanes (bottom panels). Blots shown are representative of three independent experiments. B. Quantification of Rpb1/Pgk1 ratio from three independent experiments. Error bars indicate +/− standard deviation of the mean. The anti-Rpb1 antibody has a high background, and subtraction of this background caused standard deviations to generally increase, so both background subtracted and unsubtracted quantifications are shown. (TIF) [file pgen.1002627.s006.tif]

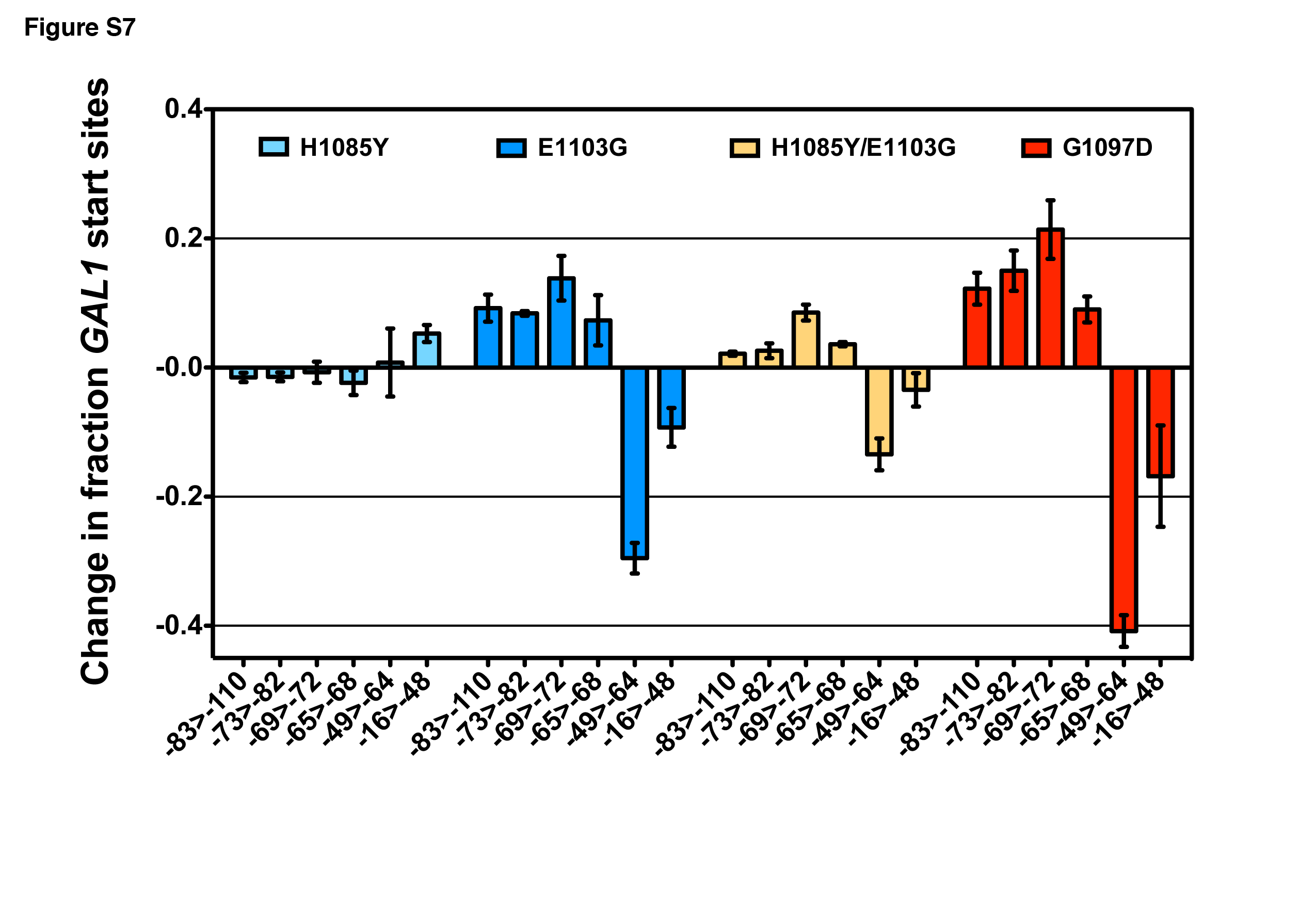

Supplement: Figure S7 — Quantification of start site distribution at GAL1 in Pol II mutants. Start site usage at GAL1 as determined by primer extension with radiolabeled oligo was divided into bins based on radioactive signal at positions of start sites relative to GAL1 ATG sequence (A is +1) and normalized to total signal per lane. WT start site fractions were subtracted from mutant start site fractions for particular regions to determine the relative alteration in start site distribution in Pol II mutant strains. A negative value indicates that the mutant has relatively lower usage for that particular group of start sites and a positive value indicates a relatively higher usage for that particular group of start sites. Values shown are the average of three independent experiments +/− standard deviation. (TIF) [file pgen.1002627.s007.tif]

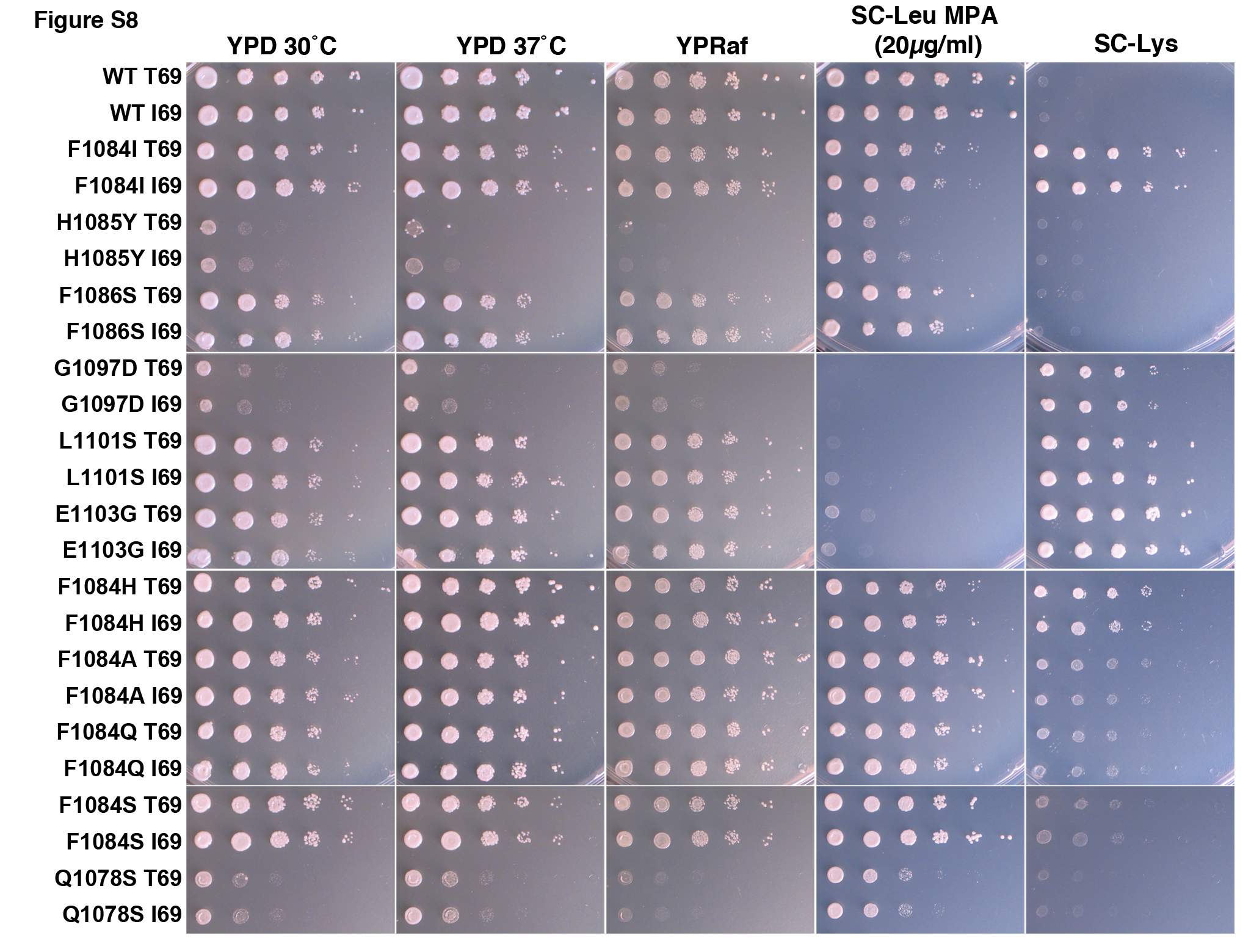

Supplement: Figure S8 — No effect of Rpb1 I69 substitution on in vivo phenotypes. 10-fold serial dilutions of yeast strains expressing T69 or I69 variants of Rpb1 as the sole source of Rpb1 were spotted onto various media to determine phenotypes. Results indicate that I69 is phenotypically inert. (TIF) [file pgen.1002627.s008.tif]
