# Supplementary material for: Dissection of Pol II Trigger Loop Function and Pol II Activity–Dependent Control of Start Site Selection In Vivo
Source: PLoS Genet. 2012 Apr 12;8(4):e1002627. doi: 10.1371/journal.pgen.1002627 (PMC3325174; doi:10.1371/journal.pgen.1002627)
Supplement: Table S1 — rpb1 mutant plasmids used in genetic analyses of the Pol II TL. (DOCX) [file pgen.1002627.s009.docx]

**Table S1**

| **Mutant** | **I69 Plasmid** | **T69 Plasmid** | **I69 Reference** | **Phenotype/Note** |
| --- | --- | --- | --- | --- |
| WT (URA3) | pCK518 | - |  | Viable. XhoI-SacI fragment containing *RPB1* from pCK347 (a pRS316 [[2](#_ENREF_2)] derivative), which contains a *HinDIII* fragment containing *RPB1* derived from a pRS314-derivative [[10](#_ENREF_10)] of pRP112 [[9](#_ENREF_9)]. |
| WT | pCK503 | pCK859 |  | Viable |
| A1076T | pCK743 | pCK968 |  | Viable |
| Q1078A | pCK799 | pCK935 |  | Inviable |
| Q1078N | pCK519 | pCK860 | [[5](#_ENREF_5)] | Inviable |
| Q1078S | pCK805 (recloned) | pCK863 | Original I69 Q1078S plasmid studied in [[5](#_ENREF_5)] was determined to have a frameshift created during the cloning process, explaining inviability in previous study. | Viable, see adjacent note |
| M1079R | pCK568 | pCK872 |  | Viable |
| M1079T | pCK752 | pCK925 |  | Viable |
| M1079V | pCK525 | pCK865 |  | Viable |
| L1081A | pCK789 | pCK934 |  | Inviable |
| L1081G | pCK788 | pCK932 |  | Inviable |
| L1081F | pCK825 | pCK1016 |  | Inviable |
| L1081I | pCK782 | pCK966 |  | Inviable |
| N1082A | pCK637 | pCK885 |  | Inviable |
| N1082R | pCK580 | pCK874 |  | Viable |
| N1082S | pCK638 | pCK886 |  | Viable |
| F1084A | pCK587 | pCK958 | [[5](#_ENREF_5)] | Viable |
| F1084H | pCK569 | pCK873 | [[5](#_ENREF_5)] | Viable |
| F1084I | pCK579 | pCK955 | [[5](#_ENREF_5)] | Viable |
| F1084Q | pCK588 | pCK880 | [[5](#_ENREF_5)] | Viable |
| F1084S | pCK590 | pCK882 | [[5](#_ENREF_5)] | Viable |
| H1085A | pCK521 | pCK861 | [[5](#_ENREF_5)] | Inviable |
| H1085D | pCK816 | pCK943 |  | Inviable |
| H1085F | pCK570 | pCK956 | [[5](#_ENREF_5)] | Inviable |
| H1085K | - | pCK1005 |  | Viable |
| H1085N | pCK717 | pCK920 |  | Inviable |
| H1085Q | pCK639 | pCK887 |  | Viable |
| H1085R | pCK522 | pCK862 |  | Viable |
| H1085W | pCK640 | pCK888 |  | Viable |
| H1085Y | pCK534 | pCK870 | [[5](#_ENREF_5)] | Viable |
| F1086S | pCK538 | pCK871 | [[5](#_ENREF_5)] | Viable |
| F1086Y | pCK663 | pCK891 |  | Viable |
| T1095A | pCK589 | pCK881 |  | Viable |
| T1095R | - | pCK1188 |  | Viable |
| G1097D | pCK529 | pCK867 | [[5](#_ENREF_5)] | Viable |
| L1101S | pCK809 | pCK864 | [[5](#_ENREF_5)] | Viable |
| E1103G | pCK527 | pCK960 | [[5](#_ENREF_5)] | Viable |
| E1103A | pCK641 | pCK889 |  | Viable |
| N1106D | pCK609 | pCK883 |  | Viable |
| R446A | pCK705 | pCK911 | [[7](#_ENREF_7)] | Inviable |
| R446A/E1103G | pCK706 | pCK912 |  | Inviable |
| N479S | pCK362 | pCK856 | [[7](#_ENREF_7)] | Viable |
| N479S/E1103G | - | pCK964 |  | Viable |
| Q1078N/E1103G | pCK669 | pCK895 |  | Viable |
| Q1078S/E1103G | pCK670 | pCK896 |  | Viable |
| Q1078A/E1103G | pCK811 | pCK947 |  | Viable |
| L1081G/E1103G | pCK800 | pCK936 |  | Inviable |
| L1081F/E1103G | pCK803 | pCK939 |  | Viable |
| L1081A/E1103G | pCK830 | pCK948 |  | Inviable |
| L1081I/E1103G | pCK846 | pCK967 |  | Inviable |
| N1082A/E1103G | pCK671 | pCK897 |  | Viable |
| N1082S/E1103G | pCK672 | pCK898 |  | Viable |
| H1085R/E1103G | pCK676 | pCK902 |  | Viable |
| F1084I/E1103G | pCK834 | pCK952 |  | Inviable |
| H1085Y/E1103G | pCK642 | pCK890 |  | Viable |
| H1085A/E1103G | pCK673 | pCK899 |  | Viable |
| H1085F/E1103G | pCK674 | pCK900 |  | Viable |
| H1085Q/E1103G | pCK675 | pCK901 |  | Viable |
| H1085W/E1103G | pCK677 | pCK903 |  | Viable |
| F1086S/E1103G | pCK710 | pCK914 |  | Viable |
| G1097D/E1103G | - | pCK1189 |  | Inviable |
| N445Y/H1085Y | pCK585 | pCK879 |  | Inviable |
| ∆TL Tip/E1103G | pCK709 | pCK913 |  | Inviable |
| N479S/Q1078S/E1103G | pCK758 | pCK931 |  | Viable |
| N1082S/H1085Y/E1103G | pCK685 | pCK905 |  | Viable |
| N1082A/H1085Y/E1103G | pCK686 | pCK906 |  | Inviable |
| N1082A/H1085A/E1103 | pCK690 | pCK908 |  | Inviable |
| Q1078S/N1082A/E1103G | pCK691 | pCK909 |  | Viable |
| Q1078S/H1085A/E1103G | - | pCK1187 |  | Viable |
| N479S/N1082A/E1103G | - | pCK1161 |  | Inviable |
| N479S/H1085A/E1103G | - | pCK963 |  | Viable |
| Q1078A/H1085Y/E1103G | pCK801 | pCK937 |  | Inviable |
| Q1078A/H1085A/E1103 | pCK802 | pCK938 |  | Inviable |
| Q1078A/N1082A/E1103G | pCK828 | pCK946 |  | Viable |
| F1084I/H1085Y/E1103G | - | pCK1002 |  | Inviable |
| N1082A/F1084I/E1103G | - | pCK1003 |  | Viable |
| N1082S/F1084I/E1103G | - | pCK1004 |  | Viable |
| Q1078S/N1082A/H1085Y/E1103G | pCK711 | pCK915 |  | Viable |
| A1076V/Q1078S/N1082A/H1085Y/E1103G | pCK712 | pCK916 |  | Viable |
| A1076V/Q1078A/N1082A/H1085Y/E1103G | pCK826 | pCK944 |  | Inviable |
| N479S/H1085Y | pCK584 | pCK878 |  | Inviable |
| N479S/H1085A | pCK753 | pCK926 |  | Inviable |
| N479S/N1082A | pCK755 | pCK928 |  | Inviable |
| N479S/N1082S | pCK756 | pCK929 |  | Inviable |
| N479S/H1085Q | pCK757 | pCK930 |  | Inviable |
| N479S/F1084I | pCK806 | pCK941 |  | Viable |
| N479S/F1086S | - | pCK961 |  | Inviable |
| N479S/Q1078S/N1082A | pCK807 | pCK942 |  | Inviable |
| N479S/N1082A/E1103G | - | pCK1161 |  | Viable |
| N1082R/F1084I | pCK581 | pCK875 |  | Viable, Suppression of F1084I phenotypes |
| N1082S/H1085Y | - | pCK1185 |  | Inviable |
| N1082R/H1085Y | pCK583 | pCK877 |  | Inviable |
| Q1078S/N1082A | pCK684 | pCK904 |  | Viable |
| F1084I/H1085Y | pCK582 | pCK876 |  | Inviable |
| H1085Y/F1086S | pCK667 | pCK894 |  | Inviable |
| F1084I/H1085Q | pCK835 | pCK953 |  | Viable |
| N1082A/F1084I | pCK836 | pCK954 |  | Viable |
| H1085Y/F1086Y | pCK665 | pCK893 |  | Inviable |
| N1082A/H1085Y | pCK713 | pCK917 |  | Inviable |
| Q1078S/N1082S | - | pCK1186 |  | Viable |
| Q1078S/F1084I | - | pCK1184 |  | Viable |
| Q1078S/H1085Q | - | pCK1183 |  | Inviable |
| Q1078S/H1085Y | pCK714 | pCK918 |  | Inviable |
| Q1078S/H1085A | pCK715 | pCK919 |  | Inviable |
| Q1078A/H1085Y | pCK804 | pCK940 |  | Inviable |
| Q1078A/N1082A | pCK827 | pCK945 |  | Inviable |
| Q1078A/H1085A | pCK832 | pCK950 |  | Inviable |
| N1082S/F1084I | pCK833 | pCK951 |  | Viable |
| H1085A/G1097D | - | pCK1007 |  | Viable |
| Q1078A/G1097D | - | pCK1008 |  | Viable |
| N1082A/G1097D | - | pCK1009 |  | Viable |
| Q1078A/N1082A/H1085Y | pCK831 | pCK949 |  | Inviable |
| Q1078S/N1082A/F1084I | pCK847 | pCK1006 |  | Viable |
| N1082A/H1085A/G1097D | - | pCK1011 |  | Inviable |
| Q1078S/N1082A/H1085Y | - | pCK1012 |  | Inviable |
