# Supplementary material for: Dissection of Pol II Trigger Loop Function and Pol II Activity–Dependent Control of Start Site Selection In Vivo
Source: PLoS Genet. 2012 Apr 12;8(4):e1002627. doi: 10.1371/journal.pgen.1002627 (PMC3325174; doi:10.1371/journal.pgen.1002627)
Supplement: Table S2 — Strain genotypes for yeast used in this study. (DOCX) [file pgen.1002627.s010.docx]

**Table S2**

| **Strain Number** | **Genotype** | **Reference** |
| --- | --- | --- |
| CKY274 | *MATalpha ura3-52 his3∆200 leu2∆1 or ∆0 trp1∆63 met15∆0 lys2-128∂ gal10∆56 RPB3::TAP::KlacTRP1* |  |
| CKY283 | *MAT***a** *ura3-52 his3∆200 leu2∆1 or ∆0 trp1∆63 met15∆0 lys2-128∂ gal10∆56 rpb1∆::CLONATMX RPB3::TAP::KlacTRP1* [pRP112 *RPB1 URA3 CEN*] |  |
| CKY944 | *MAT***a** *ura3-52 his3∆200 leu2∆0 or ∆1 trp1∆63 met15∆0 lys2-128∂ rpb1∆::CLONATMX RPB3::3XFLAG::kanmx kanmx::GAL1p::YLR454w* [pCK518 *RPB1 URA3 CEN*] |  |
