# Supplementary material for: Dissection of Pol II Trigger Loop Function and Pol II Activity–Dependent Control of Start Site Selection In Vivo
Source: PLoS Genet. 2012 Apr 12;8(4):e1002627. doi: 10.1371/journal.pgen.1002627 (PMC3325174; doi:10.1371/journal.pgen.1002627)
Supplement: Text S1 — Description of plasmids, yeast strains, supporting discussion of genetic analyses of Pol II TL residues, and supporting methods. (DOCX) [file pgen.1002627.s011.docx]

**Text S1**

**Yeast plasmids and nucleic acids**

Construction of mutants by site-directed mutagenesis was performed using standard Quikchange mutagenesis (Stratagene) per manufacturer’s instructions using plasmids containing subclones of *RPB1* containing the TL region ligated into pBluescript II (Strategene). After verification of correct mutated sequence, *rpb1* mutant DNA was subcloned back into pCK859 (*RPB1* WT) or appropriate *rpb1* mutant plasmid for combinatorial mutant construction. All final constructs have been verified by sequencing. *RPB1* plasmids are listed in Table S1. A subset of mutants was identified in genetic screens (to be described elsewhere). Complete sequences and detailed description of all plasmids are available upon request.

**Yeast strains**

All yeast strains are derived from a *GAL2^+^* derivative of S288C [[1](#_ENREF_1)]. Pol II mutant plasmids on *LEU2* pRS315 based plasmids [[2](#_ENREF_2)] were transformed into CKY283 followed by replica-plating on medium containing 5-fluoroorotic acid (Toronto Research Chemical or Gold Biotechnology) to remove cells retaining the WT *RPB1* *URA3* plasmid. Strain genotypes are listed in Table S2.

**Genetic analysis of TL residues**

***Q1078 and N1082***

In general, TL NIR residues appear to act in a concerted fashion, with individual contributions by each residue to Pol II activity. Substitutions in particular residues may also alter TL conformation as unexpected suppression is observed (Q1078S suppression of N1082A even though both individual substitutions behave as LOF genetically in other combinations). The relationship between other Q1078 and N1082 substitutions indicates that these residues may have similar or related function and when one is compromised, the function of the other is compromised (they show epistasis). Possibly, Q1078 contributes to lethality when N1082A is present, and substitution of serine at 1078 can relieve this lethality. S1078 likely still has some positive function because Q1078A is lethal on its own and does not suppress N1082A. Furthermore, when analogous *Tth* RNAP residue Q1235 is compromised, transcriptional defects are observed, but no further defects are observed when Q1235A is present with R1239A/H1242A. These findings were interpreted to suggest *Tth* Q1235 does not participate in catalysis because of this result, because when direct contributions of *Tth* R1239 and H1242 are both compromised, Q1235 does not further influence activity. We find evidence in *S. cerevisiae* that Q1078 and N1082 functions are linked, and the relationships between these residues and N479 or F1084 substitutions are distinct from their relationships with H1085. Taken together, our results are consistent with *Tth* data [[3](#_ENREF_3)], but our examination of additional specific double mutants (in presence of E1103G suppression) shows that either Q1078A or N1082A can enhance defects of H1085A, Q1078 cannot be distinguished from N1082 in its relationship to H1085.

One difference between the bacterial *E. coli* and *Tth* RNAPs and *S. cerevisiae* Pol II is that these enzymes have an arginine in in place of N1082 in Pol II. It has been speculated that arginine in this position in *E. coli* might buffer effects of loss of H1085*^Eco^*^H936^ because of the additional basic sidechain of (R)1082*^Eco^*^R933^. We examined this possibility by substituting Arginine for N1082 (Figure 1). We observed weak phenotypes consistent with LOF behavior. We also observed synthetic phenotypes upon combination of N1082R with H1085Y (lethality, Table S1) or F1084I (suppression, Table S1). Taken together, these results suggest that N1082R is a weak LOF and does not compensate for loss of the H1085 basic sidechain.

***L1081***

L1081 substitutions show the strongest phenotypes of all single substitutions tested. No viable single mutant substitutions were identified among L1081F, L1081A, L1081I or L1081G, suggesting a greater sensitivity of this position to mutation (Table 1, Table S1). Furthermore, while all other lethal substitutions in NIR residues were suppressible by combination with E1103G, only L1081F was suppressible by E1103G among L1081 substitutions. These results are consistent with results for substitution of the analogous methionine in bacterial RNAPs that indicate stronger biochemical defects for alteration of this residue [[3](#_ENREF_3),[4](#_ENREF_4)].

***F1084***

F1084 is unique among NIR residues in that genetically it behaves it different fashions depending on genetic interaction partners (Table 1, Supplemental Figure 3). F1084 is not predicted to make direct contacts with NTP substrates but may be critical for appropriate conformation of folding of adjacent NIR residues. Some substitutions in F1084 are relatively mild or moderate phenotypes (F1084Q/S/A) while F1084H and F1084I show stronger phenotypes [[5](#_ENREF_5)](Figure 2). Phenotypically, due to its Spt^-^ and MPA^s^ phenotypes *in vivo*, and increased misincorporation rate *in vitro*, F1084I is classified as a GOF substitution [[5](#_ENREF_5)]. Consistent with this, combination of F1084I with other genetic and biochemical GOF substitutions, E1103G or G1097D, results in lethality, implying aggravation of single substitution defects in the double substitutions. Consistent with this supposition, F1084I/E1103G can be suppressed by additionally adding a genetically or biochemically LOF substitution to the pair (for example N479S, N1082S, or N1082A, but not H1085Y). F1084I also shows mostly suppressive relationships with N479S, Q1078 or N1082 substitutions but synthetic lethality with H1085 substitutions, though all these partners individually behave as LOF substitutions. These results are consistent with N479, Q1078 and N1082 forming a functional group distinct from H1085. We interpret these results as F1084I GOF activity requiring H1085, and upon substitution of H1085, F1084 becomes aggravating to H1085 substitutions.

***H1085***

Consistent with its proposed importance to Pol II and RNAP mechanisms [[6](#_ENREF_6),[7](#_ENREF_7)], substitution of H1085 has strong consequences for viability and Pol II activity [[5](#_ENREF_5)] (Table 1, Figure 2). This being said, the effects of H1085 are not special among NIR residues proposed to contact NTP substrates, nor do any other residues tested behave as if they function through H1085 (except F1084 in special circumstances, *e.g.* F1084I) because substitutions in other NIR residues exacerbate H1085 substitutions, either in an otherwise WT background or in the presence of E1103G. The strong defects of H1085K or H1085R substitutions are similar in strength to non-basic substitutions (H1085Y) or worse (H1085Q), suggesting that this residue may function in a fashion specific for the histidine side chain that is not compensated by a different basic group as might be imagined for this residue if it functioned strictly as a general acid in Pol II catalysis.

***E1103***

E1103G conferred superactivity is not able to completely bypass TL NIR residues. E1103G cannot suppress deletion of the TL tip (Table 1), most L1081 substitutions, R446A, or certain compound substitutions of TL NIR residues (Table S1). In addition, close comparison of E1103G and G1097D suppression of Q1078A, N1082 and H1085A phenotypes reveals that suppression of conditional plate phenotypes has some allele specificity. These data suggest that either or both E1103G and G1097D may alter more than TL dynamics but possibly also TL conformation (Figure S2).

**Determination of *in vitro* elongation rate**

Elongation rate for each Pol II enzyme were determined generally as previously described by performing *in vitro* transcription reactions over a time course with different concentrations of NTPs (at equimolar amounts of all four NTPs (GE Healthcare)) [[5](#_ENREF_5)]. Run off transcripts were quantified as fraction of total signal per lane for transcripts 10 nucleotides and above. Values are entered into GraphPad Prism 5.0 and half times for reactions calculated (the time that run off-transcription is 50% maximum). Maximal run-off for each time course/concentration was determined by non-linear regression of run-off values using either “One-phase Exponential Association” or “Plateau the Increase to Top” equations as determined by best-fit analysis in the program. Half times were used to determine elongation rates in nucleotides/second by division of transcript length above 10 nucleotides (51 nucleotides). These elongation rates were plotted versus substrate (NTP) concentration to infer a maximal elongation rate using non-linear regression in GraphPad Prism using “One-phase Exponential Association” or “One-site Binding (Hyperbola)” equations as determined by best-fit analysis in the program. We noticed that at higher NTP concentrations and for faster Pol II mutants, there appeared to be a plateau of signal for run-off transcript followed by an occasional decrease in signal for run-off transcript and the appearance of a heterogenous smear of transcripts longer than run-off length. We speculate that this may indicate template switching or jumping of Pol II stalled at the very end of the DNA template. For more accurate determination of elongation rates, longer time point values have been omitted from the analysis.

**Western Blotting for Rpb1 levels in Pol II mutant strains**

Yeast cell extracts from Pol II mutant strains were generated as described [[8](#_ENREF_8)] with the following modifications. Aliquots from saturated overnight yeast cell cultures (YPD) were amplified in 25ml YPD until the cell concentrations reached approximately 1.5x10^7^ /ml. 10ml culture was harvested by centrifugation, following treatment to cell pellet (scaled to ¾ of original protocol to concentrate extract). For 1X samples, 24µl of cell lysates were loaded and for 1/3X samples, 8µl were loaded onto 10% acrylamide gels (Next Gel, Amresco) and separated polypeptides subsequently transferred to Westran membranes for blotting. Anti-Rpb1 (y-80, sc-25758) from Santa Cruz Biotechnology was used as primary antibody for Rpb1 levels. Anti-Pgk1 monoclonal antibody from Molecular Probes/Invitrogen (459250) was used as primary antibody for the purpose of a loading control.

**Replacement of *RPB1* I69 substitution-encoding sequence with *RPB1* T69-encoding sequence**

During the course of our experiments, we discovered that *RPB1* plasmids derived from pRP112 [[9](#_ENREF_9)] contained a single difference in coding from the Yeast Genome Database sequence for *RPO21* (*RPB1*), an isoleucine at position 69 in place of a threonine. We found this substitution in our *RPB1* plasmid derived from a pRS314-based plasmid from the Ponticelli lab [[10](#_ENREF_10)]. We inferred that this plasmid was derived from an original Young Lab plasmid, pRP112. We sequenced an isolate of pRP112 and found the same T69I substitution, indicating this substitution was derived from pRP112 or a prior *RPB1* derivative. T69 is adjacent to a zinc-binding motif in Rpb1, mutation of which causes a temperature-sensitive phenotype [[11](#_ENREF_11)]. I69 substitution causes no detectable phenotypes in our assays whether by itself or in combination with any of our tested mutants (Figure S8). We consider this substitution phenotypically inert in our phenotypic assays and it has no effect on WT activity (Figures S4 and S5). pCK347 (*RPB1 LEU2 CEN*) [[5](#_ENREF_5)] was digested with NcoI and SpeI and transformed into CKY274, selecting for Leu^+^ transformants, putatively gap-repaired from the *RPB1* genomic sequence. Full-length plasmid candidates were isolated and sequenced to confirm replacement of I69 sequence with T69 sequence, creating pCK841. The NcoI-SpeI fragment form pCK841 was then used to replace I69-containing NcoI-SpeI fragments from Pol II mutant plasmids.

**Supporting References**

1. Winston F, Dollard C, Ricupero-Hovasse SL (1995) Construction of a set of convenient Saccharomyces cerevisiae strains that are isogenic to S288C. Yeast 11: 53-55.

2. Sikorski RS, Hieter P (1989) A system of shuttle vectors and yeast host strains designed for efficient manipulation of DNA in Saccharomyces cerevisiae. Genetics 122: 19-27.

3. Yuzenkova Y, Bochkareva A, Tadigotla VR, Roghanian M, Zorov S, et al. (2010) Stepwise mechanism for transcription fidelity. BMC Biol 8: 54.

4. Zhang J, Palangat M, Landick R (2010) Role of the RNA polymerase trigger loop in catalysis and pausing. Nat Struct Mol Biol 17: 99-104.

5. Kaplan CD, Larsson KM, Kornberg RD (2008) The RNA polymerase II trigger loop functions in substrate selection and is directly targeted by alpha-amanitin. Molecular cell 30: 547-556.

6. Castro C, Smidansky ED, Arnold JJ, Maksimchuk KR, Moustafa I, et al. (2009) Nucleic acid polymerases use a general acid for nucleotidyl transfer. Nat Struct Mol Biol 16: 212-218.

7. Wang D, Bushnell DA, Westover KD, Kaplan CD, Kornberg RD (2006) Structural basis of transcription: role of the trigger loop in substrate specificity and catalysis. Cell 127: 941-954.

8. von der Haar T (2007) Optimized protein extraction for quantitative proteomics of yeasts. PloS one 2: e1078.

9. Nonet M, Sweetser D, Young RA (1987) Functional redundancy and structural polymorphism in the large subunit of RNA polymerase II. Cell 50: 909-915.

10. Majovski RC, Khaperskyy DA, Ghazy MA, Ponticelli AS (2005) A functional role for the switch 2 region of yeast RNA polymerase II in transcription start site utilization and abortive initiation. J Biol Chem 280: 34917-34923.

11. Donaldson IM, Friesen JD (2000) Zinc stoichiometry of yeast RNA polymerase II and characterization of mutations in the zinc-binding domain of the largest subunit. The Journal of biological chemistry 275: 13780-13788.
